# *Fusobacterium nucleatum* drives endothelial cell senescence by disrupting NOX4/NRF2 balance

**DOI:** 10.1128/mbio.03441-25

**Published:** 2026-01-08

**Authors:** Peiyao Wu, Jieyu Zhou, Jun Wang, Yafei Wu, Hui Wang, Lei Zhao

**Affiliations:** 1 State Key Laboratory of Oral Diseases & National Center for Stomatology & National Clinical Research Center for Oral Diseases, West China Hospital of Stomatology, Sichuan University 616175 , Chengdu, China; 2 Department of Periodontics, West China School & Hospital of Stomatology, Sichuan University 168305, Chengdu, China; University of California, Davis, Davis, California, USA

**Keywords:** *Fusobacterium nucleatum*, atherosclerosis, senescence, NOX4/NRF2 balance, endothelial cell

## Abstract

Endothelial cell senescence plays a critical role in the development of atherosclerosis. *Fusobacterium nucleatum* (*Fn*), a periodontal pathogen, has demonstrated pro-atherosclerotic effects, yet its role in endothelial senescence remains underexplored. This study aimed to elucidate whether and how *Fn* drives endothelial senescence and contributes to atherosclerosis. *In vivo*, chronic *Fn* infection accelerated atherosclerotic plaque progression, increased oxidative stress and vascular senescence, and impaired endothelial function. *In vitro* studies revealed that *Fn* induces dose- and time-dependent ROS production, disrupts redox homeostasis, and promotes endothelial senescence and dysfunction. Mechanistically, early infection transiently activated NRF2 via AKT-dependent inhibition of GSK3β, enhancing antioxidant defenses, but prolonged infection suppressed NRF2 activity due to AKT dephosphorylation and GSK3β activation, sustaining NOX4 upregulation and exacerbating oxidative stress. This redox imbalance amplified oxidative stress, aggravated endothelial dysfunction, and promoted cellular senescence. Pharmacological inhibition of NOX4 or GSK3β restored redox balance, reduced senescence, and improved endothelial function. These findings highlight the NOX4/NRF2 axis as a potential therapeutic target for vascular aging and atherosclerosis associated with chronic periodontal infection.

**IMPORTANCE:**

In this study, we (i) demonstrated how *Fusobacterium nucleatum* (*Fn*) infection triggers a complex interplay between oxidative stress and antioxidant defense mechanisms in endothelial cells, highlighting the critical role of the NOX4/NRF2 axis in driving endothelial senescence; (ii) revealed that early *Fn* infection activates NRF2, leading to transient antioxidant responses, but prolonged infection leads to NRF2 degradation, increasing oxidative stress and exacerbating endothelial dysfunction; and (iii) showed that targeting NOX4 or GSK3β restores redox balance, alleviates endothelial senescence, and improves vascular function. Our findings suggest that chronic oral infections, such as those caused by *Fn*, may contribute to vascular aging and the progression of atherosclerosis, underscoring the importance of oral health in preventing systemic cardiovascular diseases. This study provides new insights into the mechanisms of microbial-driven vascular aging and identifies potential therapeutic targets for combating age-related cardiovascular diseases.

## INTRODUCTION

Periodontitis is a common chronic inflammatory disease characterized by the destruction of periodontal tissues, primarily driven by dysbiotic microbial communities and host immune responses (1). *Fusobacterium nucleatum* (*Fn*), a Gram-negative anaerobe frequently isolated from periodontal lesions, has drawn particular attention for its potential systemic effects (2, 3). Increasing evidence links periodontitis to multiple systemic diseases, including cardiovascular and metabolic disorders (4–6), although the underlying mechanisms remain incompletely understood. A prominent hypothesis suggests that pathogens from the oral cavity, such as *Fn*, may disseminate through the bloodstream, exacerbating the development or progression of other systemic diseases. Studies have demonstrated that *Fn* can enter the bloodstream, causing transient bacteremia through routine activities such as brushing, accidental gum bleeding, or dental procedures (7, 8). Importantly, both previous research and our own studies have shown that *Fn* plays a direct role in the onset and progression of atherosclerosis (9, 10). Specifically, we found that *Fn* not only invades aortic tissues but also disrupts plaque composition, promoting a destabilized phenotype characterized by heightened macrophage infiltration, enhanced M1 polarization, increased lipid accumulation, elevated apoptosis, and compromised extracellular matrix integrity (9).

Atherosclerosis is a chronic inflammatory condition where lipid, inflammatory cells, and extracellular matrix accumulate in arterial walls, forming plaques that obstruct blood flow (11). Vascular aging, driven by endothelial cell senescence, plays a crucial role in atherosclerosis. Oxidative stress is central to endothelial dysfunction and vascular aging progression (12, 13). Reactive oxygen species (ROS) are regulated by NADPH oxidase 4 (NOX4) and nuclear factor erythroid 2-related factor 2 (NRF2) (14–16). NOX4, a major ROS-generating enzyme in endothelial cells, contributes significantly to cardiovascular diseases, especially under aging and hyperlipidemic conditions (17–19). In contrast, NRF2 is a key transcription factor that counters ROS damage and maintains vascular health by activating antioxidant genes like heme oxygenase-1 (HO-1), NAD(P)H quinone dehydrogenase 1 (NQO-1), and glutamate-cysteine ligase catalytic subunit (GCLC) (20, 21). Thus, understanding the NOX4/NRF2 balance could offer insights into therapies targeting oxidative stress to prevent atherosclerosis.

Recent studies have shown that *Fn* induces senescence-like changes in gingival keratinocytes, affecting lysosomal metabolism, cell proliferation, and nuclear membrane integrity (22). Senotherapeutic drugs, such as dasatinib and quercetin, alleviate these senescence features in gingival cells (23), suggesting that targeting senescence pathways may represent a potential strategy for mitigating *Fn*-related oral diseases. However, the effect of *Fn* on endothelial cell senescence remains underexplored. We hypothesize that *Fn* may contribute to systemic diseases like atherosclerosis by inducing endothelial cell senescence through the NOX4/NRF2 pathway. Our study demonstrated that *Fn* disrupts the NOX4/NRF2 balance, exacerbating oxidative stress, endothelial dysfunction, and accelerating atherosclerosis progression. Furthermore, targeting NOX4 or enhancing NRF2 activation restored redox balance, reduced ROS production, and alleviated *Fn*-induced endothelial senescence. These findings suggest that therapeutic strategies aimed at rebalancing the NOX4/NRF2 pathway could offer effective approaches for treating atherosclerosis and preventing vascular aging.

## MATERIALS AND METHODS

### Bacterial culture

*Fn* (ATCC 25586) was cultured anaerobically on blood agar plates at 37°C for 48 h, then inoculated into brain heart infusion broth (BD Biosciences, USA) for another 48 h. *E. coli* DH5α strain was cultured overnight in Luria-Bertani (BD Biosciences) medium at 37°C with aerobic shaking. For the animal experiments, *Fn* was harvested during the logarithmic phase by centrifugation (4,000 rpm, 10 min, 4°C), washed with PBS, and resuspended in 4% carboxymethyl cellulose-PBS solution (Sigma, USA) to a final concentration of 1 × 10⁹ CFU/mL.

### Mice

Male *Apoe* knockout (*Apoe*^−/−^) mice (6 weeks old, *n* = 30) were obtained from Beijing Vital River Laboratory Animal Technology and were housed under specific pathogen-free conditions with a controlled environment at the Laboratory Animal Center of Sichuan University. The mice were kept in a temperature-controlled room with a 12-h light/dark cycle and provided with food and water *ad libitum*. The mice were randomly divided into two groups, with 15 mice per group. For the experimental protocol, mice were treated with a 0.12% chlorhexidine gluconate rinse for 7 days to standardize oral hygiene prior to oral inoculation with *Fn* (1 × 10⁹ CFU/mL) or vehicle every other day for 24 weeks. Both groups were fed a high-fat diet (HFD) throughout the study period. After 24 weeks, blood and tissue samples were collected for further analysis.

### Cell culture and co-culture model

HUVECs (ATCC, USA) were cultured in DMEM (Gibco, USA) with 10% FBS, 100 μg/mL streptomycin, and 100 U/mL penicillin at 37°C in a 5% CO_2_ atmosphere. For co-culture, HUVECs (1 × 10⁵ cells/mL) were seeded into plates, allowed to adhere overnight, and then co-cultured with *Fn* at various MOI. N-acetylcysteine (NAC; 5 mM, Macklin, China), GKT137831 (5 µM, APE-Bio, USA), or lithium chloride (LiCl; 10 mM, Macklin, China) were added for 1 h prior to infection with *Fn*. Cells and supernatants were collected at different time points (up to 72 h) for analysis. Specifically, *E. coli* DH5α was used as a Gram-negative bacterial control to compare its effects on cell senescence with *Fn* for 48 h at MOI = 100.

### Transfection of siRNA

NOX4 expression was suppressed by transfecting HUVECs with NOX4-specific siRNA (Table S1) (Tsingke, China) using TSnanofect V2 reagent (Tsingke, China). After 24 h, the medium was replaced with complete growth medium for subsequent analysis.

### Serum biochemical analysis

After 24 weeks, blood was collected, and serum was separated by centrifugation. LDL-C, total cholesterol (TC), and triglyceride (TG) were measured using enzymatic assay kits (Thermo Fisher, USA) following the manufacturer’s instructions.

### Histology and staining

Aortic root tissues were fixed in 4% paraformaldehyde (Santa Cruz Biotechnology, USA). For paraffin embedding, tissues were sectioned at 5 µm, and hematoxylin and eosin (HE) staining was performed for histological analysis. For frozen embedding, tissues were embedded in OCT compound and sectioned at 8 µm. Oil Red O staining was performed to assess lipid accumulation. Plaque area and Oil Red O-positive area were analyzed using ImageJ software.

### Assessment of ROS level

Frozen aortic tissue sections and HUVECs were stained with DCFH-DA (Beyotime) or MitoSOX Red (Beyotime) to measure ROS levels. Fluorescence was visualized using a fluorescence microscope, and ROS levels were quantified using ImageJ software. Flow cytometry analysis was performed on HUVECs to quantify intracellular ROS.

### Immunofluorescence

Frozen aortic root sections were stained with anti-β-galactosidase polyclonal antibody (1:1,000, Invitrogen, USA, #A-11132). Fluorescence was visualized and imaged using a fluorescence microscope.

### Western blot

Protein extracts from aorta tissues and HUVEC cells were prepared using RIPA buffer (Cell Signaling Technology, USA) and subjected to SDS-PAGE. Proteins were probed with antibodies against p16 (1:5,000, Proteintech, China, #81373-10-RR), p21 (1:1,000, Proteintech, #10355-1-AP), NOX4 (1:1,000, Proteintech, #14347-1-AP), NRF2 (1:1,000, Proteintech, #16396-1-AP), HO-1 (1:1,000, Huabio, China, #HA721854), NQO-1 (1:1,000, Huabio, #ET1702-50), GCLC (1:1,000, Proteintech, #ET1704-38), protein kinase B (AKT, 1:2,000, Huabio, #HA721870), p-AKT (1:5,000, Huabio, #ET1607-73), glycogen synthase kinase 3 beta (GSK3β; 1:1,000, Huabio, #ET1607-71), p-GSK3β (1:5,000, Huabio, #ET1607-60), and glyceraldehyde 3-phosphate dehydrogenase (GAPDH; 1:10,000, Huabio, #HA721136). Protein levels were analyzed by densitometry using ImageJ software.

### Enzyme-linked immunosorbent assay (ELISA)

Serum levels of ICAM-1, VCAM-1, and ET-1 were measured using ELISA kits (Lianke, China). Absorbance was measured at 450 nm, and concentrations were calculated from standard curves.

### Senescence-associated β-galactosidase (SA-β-gal) staining

SA-β-gal staining was performed on tissue sections and cells using a commercial kit (Beyotime). Images were captured using a light microscope, and SA-β-gal positive cells were quantified using ImageJ software.

### Oxidative stress measurement

Malondialdehyde (MDA) and reduced glutathione (GSH)/oxidized glutathione (GSSG) levels were measured using commercial assay kits (Beyotime) according to the manufacturer’s instructions.

### Evaluating endothelial function

Nitric oxide (NO) levels were quantified using a Griess reagent assay (Beyotime). Endothelial permeability was assessed using FITC-labeled dextran (Sigma, USA) and measured with a fluorometer.

### Bioinformatics analysis

The GSE163154 data set was obtained from the GEO database (https://www.ncbi.nlm.nih.gov/geo/). This series contains 43 carotid atherosclerotic plaque samples collected from symptomatic patients undergoing carotid endarterectomy surgery (24). Microarray transcriptional profiling was performed to compare lesions at different stages: 16 no-intraplaque hemorrhage (IPH, early-stage) samples and 27 IPH (advanced-stage) samples. Differentially expressed genes (DEGs) in the GSE163154 data set were identified using NCBI’s GEO2R tool (https://www.ncbi.nlm.nih.gov/geo/geo2r/), which applies the limma package for differential expression analysis. Genes with adjusted *P* < 0.05 and |log2 fold change| > 1 were considered significantly differentially expressed. The DEGs were visualized with volcano plots via “ggplot2” package. Aging-related genes were obtained from the CellAge database (https://genomics.senescence.info/cells/), with a total of 866 senescence-related genes (SRGs) retrieved, as shown in Table S2 (25). Overlapping genes between DEGs and SRGs were identified using the online tool *Draw Venn Diagram* (http://bioinformatics.psb.ugent.be/webtools/Venn/). GO and KEGG enrichment analyses were conducted based on the DAVID database results and processed in R (version 4.4.1), with visualization performed using the ggplot2 package. For GO analysis, the top 10 enriched terms in biological process (BP), cellular component (CC), and molecular function (MF) categories were displayed, while KEGG pathway enrichment results were presented as bubble plots, and pathways with *P* < 0.05 were considered statistically significant. Protein-protein interaction (PPI) networks were constructed using the STRING database with a combined score > 0.4 and visualized in Cytoscape software (version 3.10.2). Core genes were identified using the cytoHubba plug-in in Cytoscape with six ranking algorithms (MCC, MNC, Degree, Closeness, Radiality, and EPC), and a random forest model in R was applied to the core gene set to evaluate variable importance using both MeanDecreaseGini (MDG) and MeanDecreaseAccuracy (MDA) metrics. These two complementary measures were used to rank the genes based on their importance.

### Quantitative real-time PCR (qRT-PCR)

RNAiso Plus (Takara, China) was used to extract total RNA, and TIANscript RT Kit (Tiangen, China) was used to obtain cDNA. According to the instructions of SYBR Premix Ex Taq II (Takara), qRT-PCR was conducted on PCR system. Relative expression was normalized by GAPDH and calculated using the 2-DeltaDeltaCt method. The primer sequences are listed in Table S1.

### Statistical analyses

Data are presented as mean ± SD. Statistical significance was determined using Student’s *t*-test, Mann-Whitney *U*-test, or one-way ANOVA with appropriate *post hoc* tests. *P* values <0.05 were considered statistically significant.

## RESULTS

### Periodontal chronic colonization with *Fn* exacerbates aortic oxidative stress and accelerates vascular senescence

**Fig 1:**
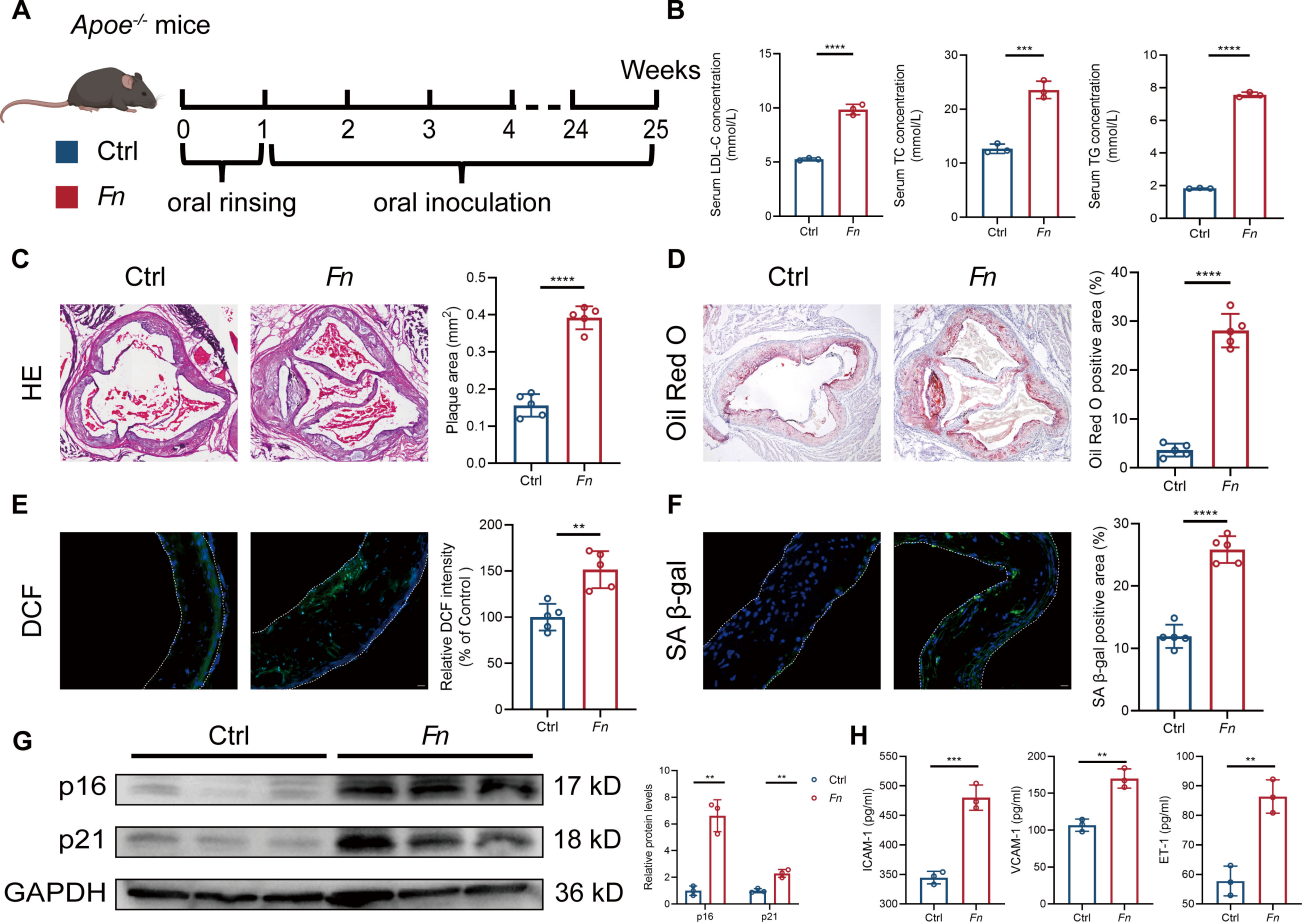
Chronic periodontal exposure to *Fn* exacerbates atherosclerosis with elevated vascular senescence and oxidative stress. (**A**) *Apoe*^−/−^ mice were fed with HFD and orally inoculated with either *Fn* (1 × 10⁹ CFU, “*Fn*” group) or vehicle (“Ctrl” group) every other day for 24 weeks. (**B**) Bar graphs showing serum levels of LDL-C (left), TC (middle), and TG (right) in the Ctrl and *Fn* groups after 24 weeks (*n* = 3 samples/group). (**C**) Representative images of HE staining in aortic root sections from the Ctrl and *Fn* groups. Bar graphs (right) show quantification of plaque area in aortic root sections (*N*=5 samples/group). Scale bars = 50 µm. (**D**) Representative images of Oil Red O staining in aortic root sections from the Ctrl and *Fn* groups with enlarged views highlighting lipid deposition (left). Bar graphs (right) show Oil Red O-positive area in aortic root sections (*n* = 5 samples/group). Scale bars = 50 µm. (**E**) Representative fluorescence images of DCF (green) and DAPI (blue) staining in aortic root sections from the Ctrl and *Fn* groups. The outline of the aorta is highlighted with a white dashed line. Bar graph (right) shows the relative DCF fluorescence intensity, normalized to the Ctrl group (*n* = 5 samples/group). Scale bars = 20 µm. (**F**) Representative immunofluorescence images of SA-β-gal (green) and DAPI (blue) staining in aortic root sections from the Ctrl and *Fn* groups. The outline of the aorta is highlighted with a white dashed line. Bar graph (right) shows the percentage of SA-β-gal-positive area, calculated as the ratio of SA-β-gal (green) area to DAPI (blue) area (*n* = 5 samples/group). Scale bars = 20 µm. (**G**) Representative western blot images (left) and quantification (right) of the protein expression levels of p16 and p21 in aortic tissues (*n* = 3 samples/group). GAPDH was used as a loading control. (**H**) Serum levels of ICAM-1 (left), VCAM-1 (middle), ET-1 (right) in the Ctrl and *Fn* groups (*n* = 3 samples/group). Data are presented as mean ± SD. **P < 0.01, ***P < 0.001, and ****P < 0.0001. ns, not significant (unpaired *t*-test).

When fed an HFD, mice with *Apoe* knockout (*Apoe^−/−^*) develop a spectrum of phenotypes closely mimicking human cardiovascular disease progression, making them a widely used model for studying cardiovascular disease (26). To investigate the role of *Fn* in cardiovascular disease, *Apoe^−/−^* mice were subjected to *Fn* infection through oral inoculation every other day for 24 weeks as previously described (27), following an initial week of antibiotic treatment and oral rinsing, while the control mice received the same HFD without *Fn* inoculation (Fig. 1A). After 24 weeks, the *Fn* group showed significantly higher levels of LDL-C, TC, and TG (Fig. 1B). HE staining revealed increased atherosclerotic plaque formation in the aortic root, with larger plaque areas (Fig. 1C), confirmed by Oil Red O staining showing enhanced lipid deposition (Fig. 1D). ROS levels were elevated in the aortas of *Fn*-infected mice (Fig. 1E), and mitochondrial ROS also increased (Fig. S1A). SA β-gal staining showed larger positive areas (Fig. 1F), and western blot indicated increased p16 and p21 expression (Fig. 1G). Endothelial dysfunction markers, including ICAM-1, VCAM-1, and ET-1, were elevated (Fig. 1H), suggesting that chronic *Fn* infection accelerates atherosclerosis, oxidative stress, and vascular aging, particularly in endothelial cells.

### *Fn* promotes endothelial cell senescence and dysfunction in a ROS-dependent manner

**Fig 2:**
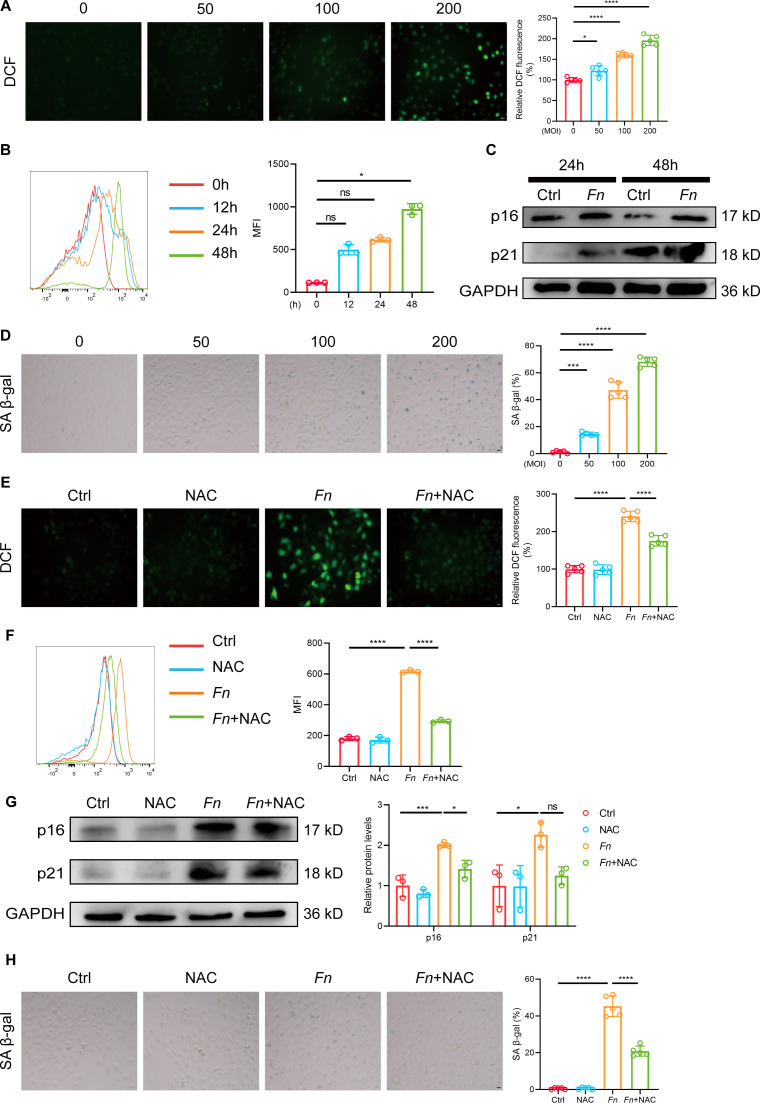
*Fn* promotes endothelial cell senescence in ROS-dependent manner. HUVECs were infected with *Fn* at varying MOIs for the indicated times (24 or 48 h). (**A**) Representative fluorescence images (left) and quantification (right) show intracellular ROS levels detected by DCFH-DA staining (*n* = 5 samples/group). Scale bar = 20 μm. (**B**) Representative histograms (left) and quantification (right) of ROS levels in HUVECs at indicated times post-infection by FACS (*n* = 3 samples/group). (**C**) Western blot analysis of senescence markers p16 and p21 in HUVECs at 24 and 48 h post-infection. GAPDH was used as a loading control. (**D**) Representative images (left) and quantification of SA-β-gal positive cells (right) in HUVECs infected with *Fn* at different MOIs (*n* = 5 samples/group). Scale bar = 20 μm. (**E–H**) HUVECs were pretreated with the ROS scavenger NAC before infection with *Fn* (MOI = 100) for 48 h. (**E**) Representative fluorescence images (left) and quantification of DCFH-DA staining intensity (right) show ROS levels at 48 h post-infection (*n* = 5 samples/group). Scale bar = 20 μm. (**F**) Representative histograms (left) and quantification (right) of ROS levels in HUVECs by FACS (*n* = 3 samples/group). (**G**) Representative western blot images (left) and quantification (right) of the protein expression levels of p16 and p21 (*n* = 3 samples/group). GAPDH was used as a loading control. (**H**) Representative images (left) and quantification of SA-β-gal-positive cells (right) in HUVECs with or without NAC (*n* = 5 samples/group). Scale bar = 20 μm. Data are presented as mean ± SD. *P < 0.05, ***P < 0.001, ****P < 0.0001. ns, not significant (one-way ANOVA with Tukey’s multiple comparison test).

*In vitro* experiments using HUVECs showed a concentration-dependent increase in ROS levels after *Fn* infection, confirmed by DCF staining (Fig. 2A) and FACS analysis (Fig. 2B). Oxidative stress was further evidenced by increased MDA levels and a reduced GSH/GSSG ratio (Fig. S1B). *Fn* infection upregulated senescence markers p16 and p21 (Fig. 2C) and induced a dose-dependent increase in SA-β-gal-positive cells (Fig. 2D). To exclude the general effects of Gram-negative bacteria, we used *E. coli* DH5α as a control. The results demonstrated that DH5α did not significantly induce cell senescence, whereas *Fn* treatment exhibited a marked pro-senescence effect (Fig. S1C and D). Furthermore, *Fn* infection also led to an increase in NO production at 24 h and a decrease at 48 h, while simultaneously causing increased endothelial permeability (Fig. S1E and F).

To investigate the role of ROS, we treated *Fn*-infected HUVECs with NAC, a potent ROS scavenger. As expected, NAC treatment significantly reduced intracellular ROS levels (Fig. 2E and F) and alleviated oxidative stress, as indicated by decreased MDA content (Fig. S1G). At the molecular level, NAC significantly attenuated the *Fn*-induced upregulation of the key senescence markers p16 (Fig. 2G). Consistently, the percentage of SA-β-gal-positive senescent cells was also markedly reduced by NAC (Fig. 2H). Furthermore, NAC treatment restored NO production and improved endothelial barrier function, thereby mitigating the impairments induced by *Fn* infection (Fig. S1H and I). Collectively, these results demonstrate that *Fn* promotes endothelial senescence and dysfunction primarily in a ROS-dependent manner.

### NOX4-ROS axis mediates *Fn*-induced endothelial cell senescence

DEG analysis of the GSE163154 data set, which includes microarray profiles of 43 carotid atherosclerotic plaques from early- and advanced-stage lesions, identified 519 DEGs (Table S3). By intersecting these DEGs with 866 SRGs from the CellAge database, 33 senescence-related differentially expressed genes (SRDEGs) were obtained (Fig. S2A and B). GO enrichment analysis revealed significant enrichment in ROS-related pathways, including superoxide metabolic process, superoxide anion generation, and superoxide-generating NAD(P)H oxidase activity (Fig. S2C). KEGG pathway analysis highlighted lipid and atherosclerosis pathways (Fig. S2D). Based on interaction information from the STRING database, 114 PPIs were mapped for the SRDEGs under a combined score threshold of >0.4 (Fig. S2E). Genes in the PPI network were ranked according to degree (Fig. S2F). To identify significant nodes and hub genes, the cytoHubba plug-in of Cytoscape was applied. Six common algorithms (MCC, MNC, Degree, Closeness, Radiality, and EPC) were used to evaluate and select hub SRDEGs, and the intersection of the top 10 genes from each algorithm yielded eight hub genes (Fig. S2G; Table S4). These eight hub genes were analyzed using a random forest model to assess their relative importance, utilizing both MDG and MDA metrics. The relationship between the number of trees and model error was visualized (Fig. S2H), and the eight genes ranked by their importance were CTSB, NOX4, MMP9, CYBB, CAV1, SOD2, CCL2, and PPARG (Fig. S2I). Among these, NOX4, a constitutive NADPH oxidase that generates superoxide intracellularly through complex formation with CYBA/p22phox and regulates signaling cascades likely via phosphatase inhibition, was selected for further investigation owing to its central role in ROS production and its higher expression relative to other NOX family members in endothelial cells (28).

**Fig 3:**
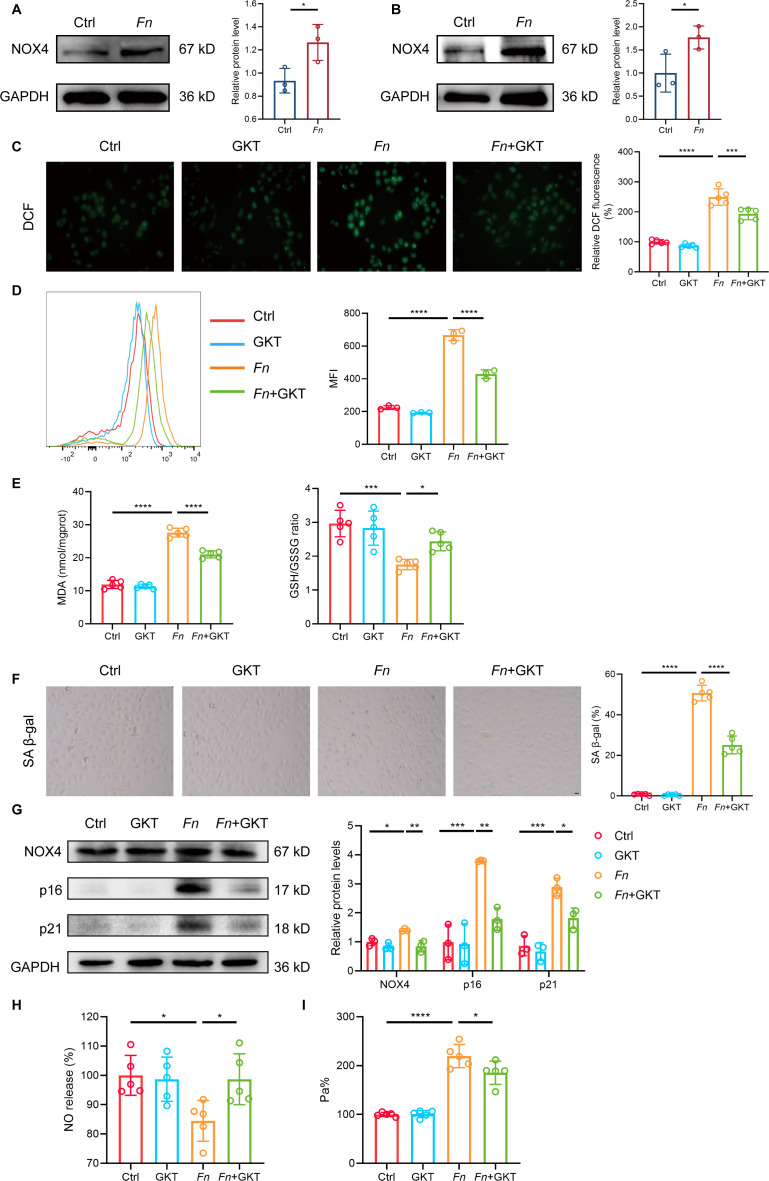
*Fn*-induced endothelial senescence is mediated by NOX4-driven ROS production. *Apoe*^−/−^ mice were fed with HFD and orally inoculated with either *Fn* (1 × 10^9^ CFU, “*Fn*“ group) or vehicle (“Ctrl” group) every other day for 24 weeks, (**A**) Representative western blot images (left) and quantification (right) of the protein expression level of NOX4 in aortic tissues (*n* = 3 samples/group). GAPDH was used as a loading control. HUVECs were infected with *Fn* for 48 h at MOI = 100, (**B**) Representative western blot images (left) and quantification (right) of the protein expression level of NOX4 in HUVECs (*n* = 3 samples/group). GAPDH was used as a loading control. (**C–I**) HUVECs were infected with *Fn* for 48 h at MOI = 100 with or without GKT137831. (**C**) Representative fluorescence images (left) and quantification (right) of intracellular ROS levels in HUVECs (*n* = 5 samples/group). Scale bar = 20 μm. (**D**) Representative histograms (left) and quantification (right) of ROS levels in HUVECs by FACS (*n* = 3 samples/group). (**E**) Quantification of MDA levels (left) and the GSH/GSSG ratio (right) (*n* = 5 samples/group). (**F**) Representative images (left) and quantification (right) of SA-β-gal staining in HUVECs (*n* = 5 samples/group). Scale bar = 20 μm. (**G**) Representative western blot images (left) and quantification (right) of the protein expression levels of NOX4, p16, and p21 in HUVECs (*n* = 3 samples/group). GAPDH was used as a loading control. (**H**) Quantification of NO (*n*=5 samples/group) and (**I**) endothelial permeability (*n* = 5 samples/group). Data are presented as mean ± SD. *P < 0.05, **P < 0.01, ***P < 0.001, and ****P < 0.0001. ns, not significant (unpaired *t*-test for panels **A and B**; one-way ANOVA with Tukey’s multiple comparison test for panels **C–I**).

We next investigated the role of NOX4 in *Fn*-induced endothelial dysfunction. Western blot analysis confirmed that *Fn* infection upregulated NOX4 expression in both aortic tissues and endothelial cells (Fig. 3A and B). The elevated ROS levels induced by *Fn* were significantly attenuated by either NOX4 inhibition (Fig. 3C and D) or gene silencing (Fig. S3A and B). Furthermore, NOX4 inhibition (Fig. 3E) or silencing (Fig. S3C) significantly reversed the *Fn*-induced alterations in MDA levels and GSH/GSSG ratio. Functional analyses demonstrated that NOX4 blockade ameliorated cellular senescence and endothelial dysfunction. SA-β-gal staining revealed that both NOX4 inhibition (Fig. S3F) and knockdown (Fig. S3D) significantly reduced the proportion of senescent cells. Consistently, Western blot analysis showed that NOX4 inhibition significantly suppressed the expression of senescence markers p16 and p21 (Fig. 3G), with a similar trend observed following NOX4 gene silencing (Fig. S3E). Additionally, both NOX4 inhibition (Fig. 3H) and gene silencing (Fig. S3F) restored NO production that had been suppressed by *Fn* infection and reduced endothelial permeability (Fig. 3I, Fig. S3G). These findings indicate that *Fn* promotes endothelial senescence and dysfunction through the NOX4-ROS axis.

### *Fn* induces an imbalance of the NOX4/NRF2 pathway in endothelial cells

**Fig 4:**
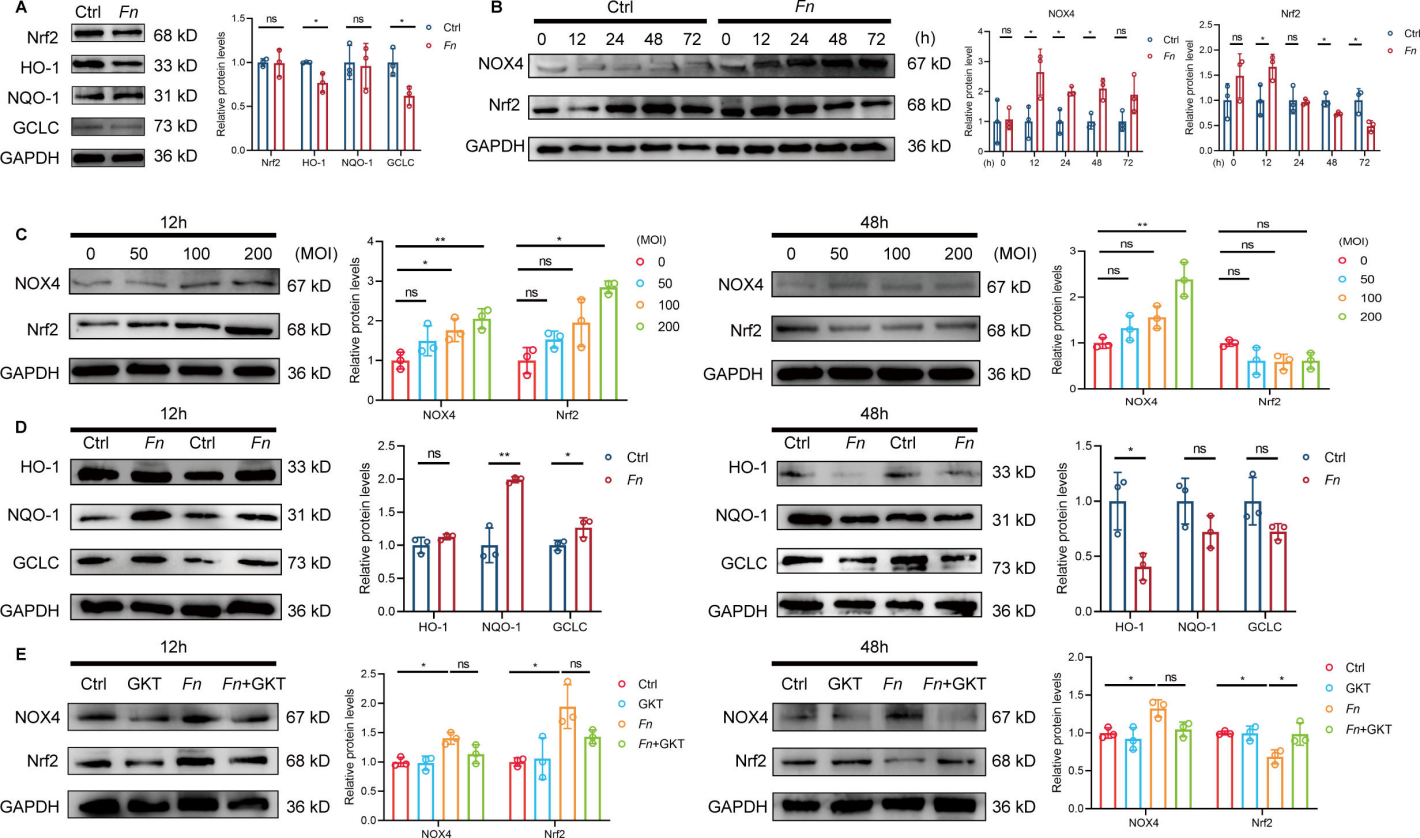
*Fn* disrupts NOX4-NRF2 redox homeostasis in endothelial cells. *Apoe*^−/−^ mice were fed with HFD and orally inoculated with either *Fn* (1 × 10^9^ CFU, “*Fn*” group) or vehicle (“Ctrl” group) every other day for 24 weeks. (**A**) Representative western blot images (left) and quantification (right) of NRF2, HO-1, NQO-1, and GCLC protein expression in aortic tissues (*n* = 3 samples/group). GAPDH was used as a loading control. HUVECs were infected with *Fn* for the indicated time at MOI = 100. (**B**) Representative western blot images (left) and quantification (right) of NOX4 and NRF2 protein expression in HUVECs (*n* = 3 samples/group). GAPDH was used as a loading control. HUVECs were infected with *Fn* at indicated MOI for 12 and 48 h. (**C**) Representative western blot images (left) and quantification (right) of NOX4 and NRF2 protein expression at 12 and 48 h post-infection (*n* = 3 samples/group). GAPDH was used as a loading control. HUVECs were infected with *Fn* at MOI = 100 for 12 and 48 h. (**D**) Representative western blot analysis (left) and quantification (right) of the NRF2 downstream antioxidant proteins HO-1, NQO-1, and GCLC in HUVECs (*n* = 3 samples/group). GAPDH was used as a loading control. HUVECs were infected with *Fn* at MOI = 100 for 12 and 48 h with or without GKT137831. (**E**) Representative western blot analysis (left) and quantification (right) of NOX4 and NRF2 protein expression in HUVECs (*n* = 3 samples/group). GAPDH was used as a loading control. Data are presented as mean ± SD. *P < 0.05 and **P < 0.01. ns, not significant (unpaired *t*-test for panels **A and B**; one-way ANOVA with Tukey’s multiple comparison test for panels **C–E**).

The NRF2/ARE pathway plays a crucial role in antioxidant defense, and its balance with NOX4 is essential for maintaining cellular redox homeostasis. To investigate whether *Fn* infection disrupts this delicate balance, we first examined *Apoe*^−/−^ mice infected with *Fn* for 24 weeks. Western blot analysis revealed significant downregulation of NRF2 downstream antioxidants HO-1 and GCLC (Fig. 4A), indicating impaired NRF2 signaling *in vivo. In vitro*, HUVECs exposed to *Fn* showed time- and dose-dependent changes. NOX4 levels were upregulated at 12, 24, and 48 h post-infection, while NRF2 levels increased at 12 h, then decreased at 48 and 72 h (Fig. 4B). Dose-dependent experiments further confirmed these temporal patterns. NOX4 expression was significantly upregulated at both MOI = 100 and MOI = 200 at 12 h post-*Fn* infection. Although a statistically significant upregulation of NOX4 at 48 h was observed only at MOI = 200, a clear increasing trend was also evident at MOI = 100. As for NRF2, its expression at 12 h displayed an upward trend with increasing MOI, reaching significance at MOI = 200, and then tended to return to baseline by 48 hs (Fig. 4C). Analysis of downstream antioxidant proteins paralleled these findings. NQO-1 and GCLC were upregulated at 12 h during the compensatory phase, but HO-1 was significantly downregulated at 48 h (Fig. 4D). To establish the causal relationship between NOX4 and NRF2 dysregulation, we employed the NOX4 inhibitor GKT137831, which successfully prevented *Fn*-induced NRF2 suppression at 48 h (Fig. 4E). Collectively, these findings demonstrate that *Fn* infection induces sustained NOX4 activation coupled with transient, ultimately insufficient NRF2 responses, resulting in disrupted NOX4/NRF2 balance, accumulated oxidative stress, and endothelial dysfunction.

### *Fn* affects the NOX4/NRF2 balance through the AKT/GSK3β pathway

**Fig 5:**
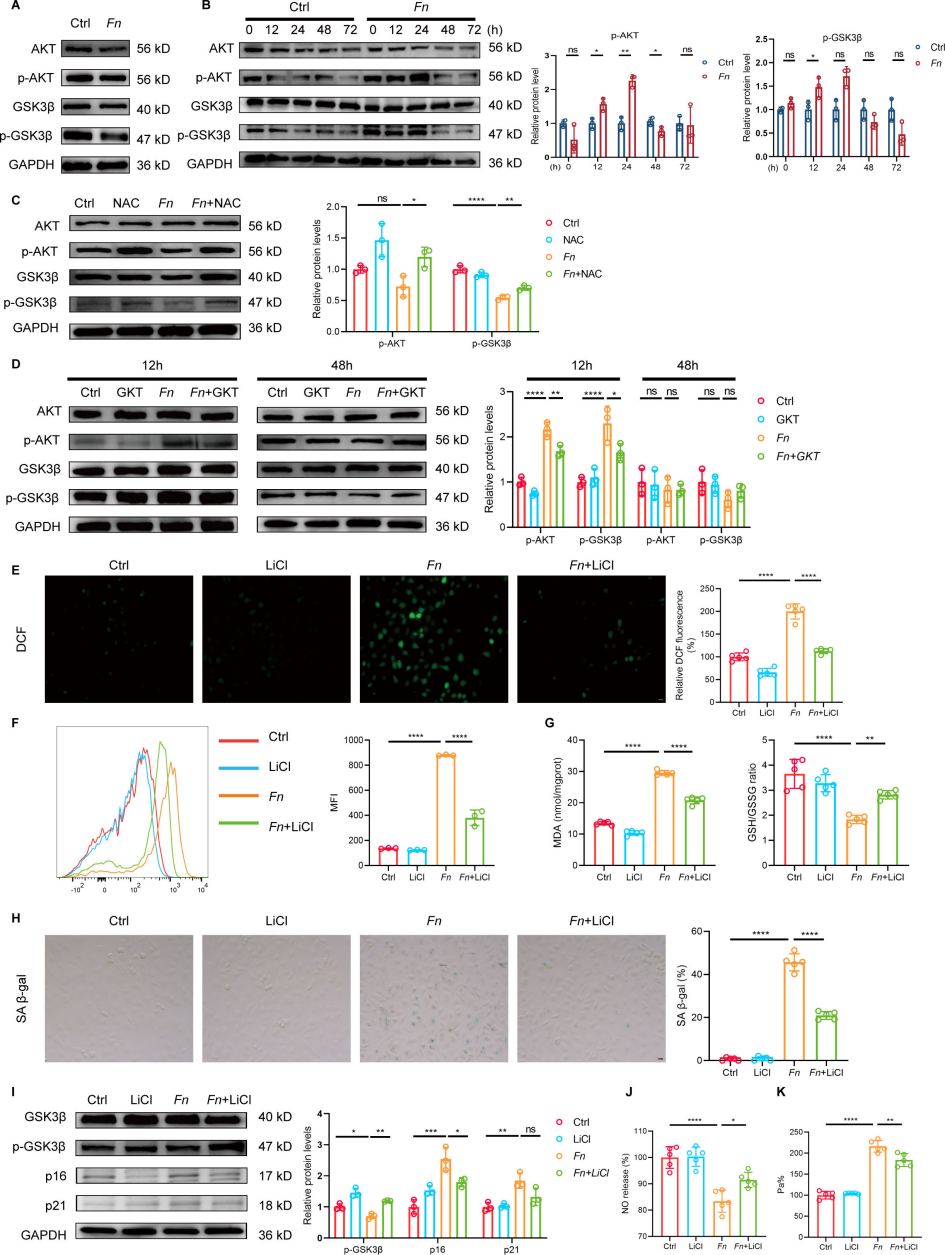
*Fn* disrupts the NOX4/NRF2 balance via the AKT/GSK3β signaling pathway. *Apoe*^−/−^ mice were fed with HFD and orally inoculated with either *Fn* (1 × 10^9^ CFU, “*Fn*” group) or vehicle (“Ctrl” group) every other day for 24 weeks. (**A**) Western blot analysis of total and phosphorylated AKT and GSK3β protein expression in aortic tissues. GAPDH was used as a loading control. HUVECs were infected with *Fn* for indicated time at MOI = 100. (**B**) Representative western blot images (left) and quantification (right) of total and phosphorylated AKT and GSK3β protein levels in HUVECs at different time points (*n* = 3 samples/group). GAPDH was used as a loading control. (**C and D**) HUVECs were infected with *Fn* for 12 and 48 h at MOI = 100 with or without NAC or GKT137831. (**C**) Representative western blot analysis (left) and quantification (right) of total and phosphorylated AKT and GSK3β protein levels in HUVECs with or without NAC (*n* = 3 samples/group). GAPDH was used as a loading control. (**D**) Representative western blot analysis (left) and quantification (right) of total and phosphorylated AKT and GSK3β protein levels in HUVECs with or without GKT137831 (*n* = 3 samples/group). GAPDH was used as a loading control. (**E–K**) HUVECs were infected with *Fn* for 48 h at MOI = 100 with or without LiCl. (**E**) Representative fluorescence images (left) and quantification (right) of intracellular ROS levels (*n* = 5 samples/group). Scale bar = 20 μm. (**F**) Representative histograms (left) and quantification (right) of ROS levels in HUVECs by FACS (*n* = 3 samples/group). (**G**) Quantification of MDA levels (left) and the GSH/GSSG ratio (right) in HUVECs (*n* = 5 samples/group). (**H**) Representative SA-β-gal staining images (left) and quantification (right) of SA-β-gal-positive cells in HUVECs (*n* = 5 samples/group). Scale bar = 20 μm. (**I**) Representative western blot analysis (left) and quantification (right) of total and phosphorylated GSK3β, p16, and p21 protein levels in HUVECs (*n* = 3). GAPDH was used as a loading control. (**J**) Quantification of NO levels (*n* = 5 samples/group) and (**K**) endothelial permeability in HUVECs (*n* = 5 samples/group). Data are presented as mean ± SD. *P < 0.05, **P < 0.01, ***P < 0.001, ****P < 0.0001. ns, not significant (unpaired *t*-test for panel **B**; one-way ANOVA with Tukey’s multiple comparison test for panels **C–K**).

To elucidate the underlying regulatory mechanisms, we first examined the transcriptional dynamics of key genes by qRT-PCR analysis. The results demonstrated that *Nox4* mRNA levels were significantly elevated at 12 and 24 h post-infection but returned to baseline by 48 and 72 h. In contrast, *Nrf2* mRNA levels remained stable across all examined time points (12, 24, 48, and 72 h) without significant fluctuations (Fig. S3H). These findings suggest that the dynamic alterations in NRF2 protein expression are not primarily driven by transcriptional regulation. Building on this observation, we further investigated the molecular mechanisms underlying NRF2 downregulation. GSK3β, a critical regulator of NRF2 stability, promotes NRF2 ubiquitination and proteasomal degradation in its active state, thereby attenuating antioxidant responses. The AKT/GSK3β signaling axis serves as a pivotal regulatory checkpoint. AKT-mediated phosphorylation inhibits GSK3β activity, whereas decreased phosphorylation leads to GSK3β hyperactivation, consequently resulting in NRF2 destabilization (29). In *Fn*-infected *Apoe^-−/^*^−^ mice, we observed significantly decreased levels of p-AKT and p-GSK3β in aortic tissues (Fig. 5A), suggesting GSK3β activation *in vivo*. Time-course analysis in HUVECs revealed that p-AKT levels were elevated at 12 and 24 h but markedly declined by 48 h post-infection (Fig. 5B). The phosphorylation pattern of GSK3β paralleled that of AKT, with significant elevation at 12 h followed by gradual return to baseline levels at later time points (Fig. 5B). To establish the causal role of oxidative stress in AKT/GSK3β dysregulation, we employed NAC, a ROS scavenger, which effectively reversed the *Fn*-induced decrease in p-AKT and p-GSK3β at 48 hs (Fig. 5C). Furthermore, NOX4 inhibition with GKT137831 prevented the early elevation of p-AKT and p-GSK3β at 12 h post-infection (Fig. 5D), establishing NOX4-derived ROS as the upstream driver of this pathway perturbation. To determine whether GSK3β activation directly contributes to endothelial dysfunction, we treated *Fn*-infected HUVECs with LiCl, a selective GSK3β inhibitor. LiCl treatment at 48 h post-infection restored NRF2 protein levels (Fig. S3I), reduced intracellular ROS accumulation (Fig. 5E and F), and alleviated oxidative stress (Fig. 5G), indicating restored redox balance. In addition, inhibition of GSK3β markedly reduced the proportion of SA-β-gal-positive senescent cells (Fig. 5H) and significantly downregulated the senescence marker p16 (Fig. 5I). LiCl treatment also rescued endothelial function by restoring NO levels (Fig. 5J) and decreasing endothelial permeability (Fig. 5K). Collectively, these results demonstrate that *Fn*-induced NOX4 activation generates sustained ROS production, which disrupts the AKT/GSK3β signaling axis, leading to GSK3β-mediated NRF2 degradation, accumulated oxidative stress, senescence, and endothelial dysfunction.

## DISCUSSION

Our study highlights the critical role of the NOX4/NRF2 axis in the pathogenesis of *Fn*-induced endothelial senescence, with a particular focus on the temporal regulation of this pathway by AKT/GSK3β signaling. We observed that in the early stages of infection, activation of the AKT/GSK3β pathway triggers NRF2 nuclear translocation, leading to the upregulation of antioxidant gene expression and a transient mitigation of oxidative stress. However, as the infection progresses, a decline in AKT phosphorylation results in the activation of GSK3β, leading to NRF2 degradation. This shift, in turn, diminishes antioxidant defenses and exacerbates NOX4-mediated ROS production, driving endothelial dysfunction and senescence. This progression mirrors the aging process in the vasculature, where a balance between oxidative stress and antioxidant defenses gradually tilts towards cellular damage and dysfunction (30).

The findings of this study underscore the importance of oxidative stress in both aging and disease. In endothelial cells, prolonged oxidative stress contributes to endothelial cell senescence, which is a central feature of vascular aging (31). Senescent endothelial cells lose their ability to respond to normal stimuli, impairing vascular homeostasis and increasing the risk of cardiovascular diseases, such as atherosclerosis, hypertension, and stroke (32, 33). These conditions are often exacerbated by chronic periodontal inflammation or periodontal pathogen infections, as seen in infections like *Fn* (34). By linking infection-induced endothelial dysfunction to aging-related processes, our study suggests that bacteria-induced ROS production may accelerate vascular aging, particularly in susceptible populations or individuals with underlying risk factors.

Intracellular ROS levels are controlled by the balance between their production and elimination. In endothelial cells, ROS are mainly produced by mitochondria and NADPH oxidases (NOX), with NOX4 being the most abundant isoform in vascular endothelial cells (35). NOX4 has a dual role in atherosclerosis; it can protect blood vessels by helping cells cope with oxidative stress (35, 36), but excessive NOX4 activity increases oxidative stress and vascular inflammation, particularly in older adults (18). Indeed, in aging mice, inhibition of NOX4 activity using GKT137831 significantly alleviates atherosclerosis (37). NOX4 also influences endothelial nitric oxide synthase uncoupling in senescent cells, exacerbating endothelial dysfunction and promoting atherosclerosis (38). In age-related endothelial dysfunction, NOX4 plays a critical role and could potentially serve as an important therapeutic target for maintaining vascular health in aging individuals (39). Our study shows that *Fn* infection increases NOX4 activity in endothelial cells, leading to cell senescence and enhanced ROS production. Reducing NOX4 or scavenging ROS alleviated oxidative stress and improved endothelial cell function, suggesting that NOX4 plays a central role in *Fn*-induced endothelial aging and its contribution to atherosclerosis.

NRF2 is a key transcription factor that regulates the cellular antioxidant response. Under normal conditions, NRF2 binds to its repressor Keap1, maintaining low expression. During oxidative stress, NRF2 dissociates from Keap1, moves to the nucleus, and activates antioxidant genes like HO-1 and NQO1. The NOX4/NRF2 axis plays a critical role in redox homeostasis. NOX4 upregulation increasing mitochondrial ROS levels, activating mitochondrial autophagy via the NRF2/PINK1 pathway (40). This coordinated response represents an adaptive mechanism for managing oxidative stress. However, this delicate balance can be disrupted by environmental insults and aging. PM2.5 exposure, for instance, elevates NOX4 expression while simultaneously suppressing NRF2 activity, leading to redox imbalance (41). Similarly, aging compromises this regulatory axis, resulting in defective mitochondrial autophagy and increased susceptibility to kidney injury (42). In aged mouse aorta, the increased expression of NOX4 was accompanied by decreased levels of NRF2, indicating a shift toward oxidative/antioxidative imbalance and contributing to vascular aging (43). Targeting the NOX4-NRF2 imbalance offers a promising therapeutic strategy to reverse age-related fibrosis and restore redox homeostasis (44). In our study, we found that targeting the NOX4/NRF2 imbalance during *Fn* infection effectively attenuated endothelial senescence and dysfunction, suggesting that modulating this redox axis could represent a novel therapeutic approach for mitigating the cardiovascular consequences of chronic periodontal infections.

Recent studies suggest that various pathogenic microorganisms can accelerate host cell senescence through different mechanisms. For instance, pathogens like Pseudomonas aeruginosa and Helicobacter pylori can secrete factors that damage host DNA or increase ROS, thereby promoting cellular senescence through activation of signaling pathways such as NF-κB and p53-p21 (45). Among periodontal pathogens, *Porphyromonas gingivalis* has been shown to induce senescence in human gingival fibroblasts through its lipopolysaccharide (46). Notably, *Fn*, a periodontal pathogen closely associated with various aging-related diseases, can induce a senescent phenotype in gingival epithelial cells, characterized by increased SA-β-gal activity, elevated γ-H2AX levels, and reduced Ki-67 expression, ultimately leading to impaired cell repair functions (22, 23). Despite these insights, the molecular mechanisms by which pathogenic microorganisms mediate host cell senescence remain incompletely understood. This study focuses on the temporal regulation of the NOX4/NRF2 axis during *Fn* infection, providing preliminary evidence that the ROS-mediated AKT/GSK3β pathway plays a key role in this process: early in the infection, activation of this pathway promotes NRF2 nuclear translocation, helping to transiently maintain redox balance; however, in later stages of infection, the pathway shifts to promote NRF2 degradation, leading to accumulated oxidative stress and induction of the senescent phenotype. Nevertheless, the full mechanism of this regulatory network requires further elucidation. Similarly, the infection process of other pathogens also displays time-dependent regulatory characteristics. For example, during *Streptococcus anginosus* infection of gastric epithelial cells, short-term infection briefly activates immune responses through the virulence proteins TMPC and annexin A2, whereas long-term infection continuously activates annexin A2 and promotes cell transformation and tumorigenesis via the MAPK/ERK/JNK pathway (47). This suggests that temporal regulation may have a universal role in microbial pathogenesis. Future studies could further explore whether *Fn* triggers similar temporal signaling processes through specific virulence factors interacting with host receptors. Furthermore, the dynamic changes in the NOX4/NRF2 axis may also be influenced by the cellular senescence state. Studies have shown that in fibroblasts, this axis exhibits age-related responses: in young cells, NOX4 activation is accompanied by upregulation of NRF2 expression; in senescent cells, sustained high expression of NOX4 leads to NRF2 downregulation, resulting in redox imbalance (44). This finding further supports the core role of the NOX4/NRF2 axis in linking infection, oxidative stress, and cellular senescence. In summary, this study provides important mechanistic insights into how bacterial infection drives vascular aging and suggests that *Fn* has the potential to serve as a key model organism for exploring the interactions between infection, oxidative stress, and vascular aging.

In conclusion, this study elucidates a novel mechanistic connection between periodontal infection and vascular atherosclerosis, demonstrating that AKT/GSK3β-mediated disruption of the NOX4/Nrf2 axis drives *Fn*-induced endothelial senescence. These findings suggest a promising therapeutic approach targeting this pathway to prevent pathogen-driven vascular dysfunction. Moreover, this work establishes a foundation for future *in vivo* validation and for exploring the broader implications of the periodontal-vascular axis in overall health.

## SUPPLEMENTAL MATERIAL

Fig. S1 mbio.03441-25-s0001.tif *Fn* induced oxidative stress, senescence, and endothelial dysfunction in HUVECs.

Fig. S2 mbio.03441-25-s0002.tif Bioinformatics analysis identifies aging-related differential genes and key regulators in atherosclerosis.

Fig. S3 mbio.03441-25-s0003.tif NOX4 silencing and LiCl treatment modulate oxidative stress and endothelial function in *Fn*-infected HUVECs.

Graphical Abstract mbio.03441-25-s0004.tif Summary.

Legends mbio.03441-25-s0005.docx Supplemental figure legends.

Supplemental blots mbio.03441-25-s0006.pdf Full uncropped WB membranes.

Table S1 mbio.03441-25-s0007.docx Primers.

Table S2 mbio.03441-25-s0008.xlsx SRGs retrieved from the CellAge Database.

Table S3 mbio.03441-25-s0009.xlsx DEGs identified from the GSE163154 data set.

Table S4 mbio.03441-25-s0010.xlsx Top 10 genes ranked by MCC, MNC, Degree, Closeness, Radiality, and EPC.

ASM does not own the copyrights to Supplemental Material that may be linked to, or accessed through, an article. The authors have granted ASM a non-exclusive, world-wide license to publish the Supplemental Material files. Please contact the corresponding author directly for reuse.

## References

[1] Hajishengallis G. 2015. Periodontitis: from microbial immune subversion to systemic inflammation. Nat Rev Immunol 15:30–44. doi:10.1038/nri3785 25534621 PMC4276050

[2] Brennan CA, Garrett WS. 2019. Fusobacterium nucleatum - symbiont, opportunist and oncobacterium. Nat Rev Microbiol 17:156–166. doi:10.1038/s41579-018-0129-6 30546113 PMC6589823

[3] Han YW. 2015. Fusobacterium nucleatum: a commensal-turned pathogen. Curr Opin Microbiol 23:141–147. doi:10.1016/j.mib.2014.11.013 25576662 PMC4323942

[4] Hajishengallis G, Chavakis T. 2021. Local and systemic mechanisms linking periodontal disease and inflammatory comorbidities. Nat Rev Immunol 21:426–440. doi:10.1038/s41577-020-00488-6 33510490 PMC7841384

[5] Li X, Wang H, Yu X, Saha G, Kalafati L, Ioannidis C, Mitroulis I, Netea MG, Chavakis T, Hajishengallis G. 2022. Maladaptive innate immune training of myelopoiesis links inflammatory comorbidities. Cell 185:1709–1727. doi:10.1016/j.cell.2022.03.043 35483374 PMC9106933

[6] Wang H, Divaris K, Pan B, Li X, Lim JH, Saha G, Barovic M, Giannakou D, Korostoff JM, Bing Y, Sen S, Moss K, Wu D, Beck JD, Ballantyne CM, Natarajan P, North KE, Netea MG, Chavakis T, Hajishengallis G. 2024. Clonal hematopoiesis driven by mutated DNMT3A promotes inflammatory bone loss. Cell 187:3690–3711. doi:10.1016/j.cell.2024.05.003 38838669 PMC11246233

[7] Fan Z, Tang P, Li C, Yang Q, Xu Y, Su C, Li L. 2023. Fusobacterium nucleatum and its associated systemic diseases: epidemiologic studies and possible mechanisms. J Oral Microbiol 15:2145729. doi:10.1080/20002297.2022.2145729 36407281 PMC9673791

[8] Martins CC, Lockhart PB, Firmino RT, Kilmartin C, Cahill TJ, Dayer M, Occhi-Alexandre IGP, Lai H, Ge L, Thornhill MH. 2024. Bacteremia following different oral procedures: systematic review and meta-analysis. Oral Dis 30:846–854. doi:10.1111/odi.14531 36750413

[9] Zhou J, Liu L, Wu P, Zhao L, Wu Y. 2022. Fusobacterium nucleatum accelerates atherosclerosis via macrophage-driven aberrant proinflammatory response and lipid metabolism. Front Microbiol 13:798685. doi:10.3389/fmicb.2022.798685 35359716 PMC8963492

[10] Zhou LJ, Lin WZ, Meng XQ, Zhu H, Liu T, Du LJ, Bai XB, Chen BY, Liu Y, Xu Y, Xie Y, Shu R, Chen FM, Zhu YQ, Duan SZ. 2023. Periodontitis exacerbates atherosclerosis through Fusobacterium nucleatum-promoted hepatic glycolysis and lipogenesis. Cardiovasc Res 119:1706–1717. doi:10.1093/cvr/cvad045 36943793

[11] Libby P. 2021. The changing landscape of atherosclerosis. Nature 592:524–533. doi:10.1038/s41586-021-03392-8 33883728

[12] Xiang Q, Tian F, Xu J, Du X, Zhang S, Liu L. 2022. New insight into dyslipidemia-induced cellular senescence in atherosclerosis. Biol Rev Camb Philos Soc 97:1844–1867. doi:10.1111/brv.12866 35569818 PMC9541442

[13] Xu S, Ilyas I, Little PJ, Li H, Kamato D, Zheng X, Luo S, Li Z, Liu P, Han J, Harding IC, Ebong EE, Cameron SJ, Stewart AG, Weng J. 2021. Endothelial dysfunction in atherosclerotic cardiovascular diseases and beyond: from mechanism to pharmacotherapies. Pharmacol Rev 73:924–967. doi:10.1124/pharmrev.120.000096 34088867

[14] He S, Wang Q, Chen L, He YJ, Wang X, Qu S. 2023. miR-100a-5p-enriched exosomes derived from mesenchymal stem cells enhance the anti-oxidant effect in a Parkinson’s disease model via regulation of Nox4/ROS/Nrf2 signaling. J Transl Med 21:747. doi:10.1186/s12967-023-04638-x 37875930 PMC10594913

[15] Sheng M, Li Q, Huang W, Yu D, Pan H, Qian K, Ren F, Luo L, Tang L. 2024. Ang-(1-7)/Mas axis ameliorates bleomycin-induced pulmonary fibrosis in mice via restoration of Nox4-Nrf2 redox homeostasis. Eur J Pharmacol 962:176233. doi:10.1016/j.ejphar.2023.176233 38043775

[16] Yang H, Chen Y, Wang Z, Huang Y, Ma Z, Zou Y, Dong J, Zhang H, Huo M, Lv M, Liu X, Zhang G, Wang S, Yang K, Zhong P, Jiang B, Kou Y, Chen Z. 2024. Dexmedetomidine affects the NOX4/Nrf2 pathway to improve renal antioxidant capacity. J Pharm Pharmacol 76:851–860. doi:10.1093/jpp/rgae044 38625054

[17] Bedard K, Krause KH. 2007. The NOX family of ROS-generating NADPH oxidases: physiology and pathophysiology. Physiol Rev 87:245–313. doi:10.1152/physrev.00044.2005 17237347

[18] Vendrov AE, Vendrov KC, Smith A, Yuan J, Sumida A, Robidoux J, Runge MS, Madamanchi NR. 2015. NOX4 NADPH oxidase-dependent mitochondrial oxidative stress in aging-associated cardiovascular disease. Antioxid Redox Signal 23:1389–1409. doi:10.1089/ars.2014.6221 26054376 PMC4692134

[19] Xu S, Chamseddine AH, Carrell S, Miller FJ Jr. 2014. Nox4 NADPH oxidase contributes to smooth muscle cell phenotypes associated with unstable atherosclerotic plaques. Redox Biol 2:642–650. doi:10.1016/j.redox.2014.04.004 24936437 PMC4052526

[20] Mimura J, Itoh K. 2015. Role of Nrf2 in the pathogenesis of atherosclerosis. Free Radic Biol Med 88:221–232. doi:10.1016/j.freeradbiomed.2015.06.019 26117321

[21] Yamamoto M, Kensler TW, Motohashi H. 2018. The KEAP1-NRF2 system: a thiol-based sensor-effector apparatus for maintaining redox homeostasis. Physiol Rev 98:1169–1203. doi:10.1152/physrev.00023.2017 29717933 PMC9762786

[22] Albuquerque-Souza E, Shelling B, Jiang M, Xia XJ, Rattanaprukskul K, Sahingur SE. 2024. Fusobacterium nucleatum triggers senescence phenotype in gingival epithelial cells. Mol Oral Microbiol 39:29–39. doi:10.1111/omi.12432 37718958 PMC10939983

[23] Rattanaprukskul K, Xia XJ, Hysa M, Jiang M, Hung M, Suslavich SF, Sahingur SE. 2025. Dasatinib and quercetin limit gingival senescence, inflammation, and bone loss. J Dent Res 104:419–427. doi:10.1177/00220345241299789 39797437 PMC11909784

[24] Jin H, Goossens P, Juhasz P, Eijgelaar W, Manca M, Karel JMH, Smirnov E, Sikkink CJJM, Mees BME, Waring O, van Kuijk K, Fazzi GE, Gijbels MJJ, Kutmon M, Evelo CTA, Hedin U, Daemen MJAP, Sluimer JC, Matic L, Biessen EAL. 2021. Integrative multiomics analysis of human atherosclerosis reveals a serum response factor-driven network associated with intraplaque hemorrhage. Clin Transl Med 11:e458. doi:10.1002/ctm2.458 34185408 PMC8236116

[25] Avelar RA, Ortega JG, Tacutu R, Tyler EJ, Bennett D, Binetti P, Budovsky A, Chatsirisupachai K, Johnson E, Murray A, Shields S, Tejada-Martinez D, Thornton D, Fraifeld VE, Bishop CL, de Magalhães JP. 2020. A multidimensional systems biology analysis of cellular senescence in aging and disease. Genome Biol 21:91. doi:10.1186/s13059-020-01990-9 32264951 PMC7333371

[26] Zhang SH, Reddick RL, Piedrahita JA, Maeda N. 1992. Spontaneous hypercholesterolemia and arterial lesions in mice lacking apolipoprotein E. Science 258:468–471. doi:10.1126/science.1411543 1411543

[27] Wu P, Bie M, Zhou J, Wang J, Zhao L. 2024. Periodontal pathogen Fusobacterium nucleatum infection accelerates hepatic steatosis in high-fat diet-fed ApoE knockout mice by inhibiting Nrf2/Keap1 signaling. J Periodontal Res 59:1220–1233. doi:10.1111/jre.13278 38795023

[28] Kim J, Seo M, Kim SK, Bae YS. 2016. Flagellin-induced NADPH oxidase 4 activation is involved in atherosclerosis. Sci Rep 6:25437. doi:10.1038/srep25437 27146088 PMC4857127

[29] Dai C, Yusuf A, Sun H, Shu G, Deng X. 2021. A characterized saponin extract of Panax japonicus suppresses hepatocyte EMT and HSC activation in vitro and CCl_4_-provoked liver fibrosis in mice: roles of its modulatory effects on the Akt/GSK3β/Nrf2 cascade. Phytomedicine 93:153746. doi:10.1016/j.phymed.2021.153746 34634746

[30] Ungvari Z, Tarantini S, Donato AJ, Galvan V, Csiszar A. 2018. Mechanisms of vascular aging. Circ Res 123:849–867. doi:10.1161/CIRCRESAHA.118.311378 30355080 PMC6248882

[31] Csiszar A, Ungvari Z, Edwards JG, Kaminski P, Wolin MS, Koller A, Kaley G. 2002. Aging-induced phenotypic changes and oxidative stress impair coronary arteriolar function. Circ Res 90:1159–1166. doi:10.1161/01.res.0000020401.61826.ea 12065318

[32] Han Y, Kim SY. 2023. Endothelial senescence in vascular diseases: current understanding and future opportunities in senotherapeutics. Exp Mol Med 55:1–12. doi:10.1038/s12276-022-00906-w 36599934 PMC9898542

[33] Minamino T, Komuro I. 2007. Vascular cell senescence: contribution to atherosclerosis. Circ Res 100:15–26. doi:10.1161/01.RES.0000256837.40544.4a 17204661

[34] Lee HR, Jun HK, Kim HD, Lee SH, Choi BK. 2012. Fusobacterium nucleatum GroEL induces risk factors of atherosclerosis in human microvascular endothelial cells and ApoE^-/-^ mice. Mol Oral Microbiol 27:109–123. doi:10.1111/j.2041-1014.2011.00636.x 22394469

[35] Schröder K, Zhang M, Benkhoff S, Mieth A, Pliquett R, Kosowski J, Kruse C, Luedike P, Michaelis UR, Weissmann N, Dimmeler S, Shah AM, Brandes RP. 2012. Nox4 is a protective reactive oxygen species generating vascular NADPH oxidase. Circ Res 110:1217–1225. doi:10.1161/CIRCRESAHA.112.267054 22456182

[36] Gray SP, Di Marco E, Kennedy K, Chew P, Okabe J, El-Osta A, Calkin AC, Biessen EAL, Touyz RM, Cooper ME, Schmidt HHHW, Jandeleit-Dahm KAM. 2016. Reactive oxygen species can provide atheroprotection via NOX4-dependent inhibition of inflammation and vascular remodeling. Arterioscler Thromb Vasc Biol 36:295–307. doi:10.1161/ATVBAHA.115.307012 26715682

[37] Vendrov AE, Lozhkin A, Hayami T, Levin J, Silveira Fernandes Chamon J, Abdel-Latif A, Runge MS, Madamanchi NR. 2024. Mitochondrial dysfunction and metabolic reprogramming induce macrophage pro-inflammatory phenotype switch and atherosclerosis progression in aging. Front Immunol 15:1410832. doi:10.3389/fimmu.2024.1410832 38975335 PMC11224442

[38] Lee HY, Zeeshan HMA, Kim HR, Chae HJ. 2017. Nox4 regulates the eNOS uncoupling process in aging endothelial cells. Free Radic Biol Med 113:26–35. doi:10.1016/j.freeradbiomed.2017.09.010 28916474

[39] Kwon OS, Noh SG, Park SH, Andtbacka RHI, Hyngstrom JR, Richardson RS. 2023. Ageing and endothelium‐mediated vascular dysfunction: the role of the NADPH oxidases. J Physiol (Lond) 601:451–467. doi:10.1113/JP283208 36416565 PMC9898184

[40] Peng C, Li X, Ao F, Li T, Guo J, Liu J, Zhang X, Gu J, Mao J, Zhou B. 2023. Mitochondrial ROS driven by NOX4 upregulation promotes hepatocellular carcinoma cell survival after incomplete radiofrequency ablation by inducing of mitophagy via Nrf2/PINK1. J Transl Med 21:218. doi:10.1186/s12967-023-04067-w 36964576 PMC10039571

[41] Fan X, Dong T, Yan K, Ci X, Peng L. 2023. PM2.5 increases susceptibility to acute exacerbation of COPD via NOX4/Nrf2 redox imbalance-mediated mitophagy. Redox Biol 59:102587. doi:10.1016/j.redox.2022.102587 36608590 PMC9813701

[42] Hu J, Hou W, Ma N, Zhang Y, Liu X, Wang Y, Ci X. 2024. Aging-related NOX4-Nrf2 redox imbalance increases susceptibility to cisplatin-induced acute kidney injury by regulating mitophagy. Life Sci 336:122352. doi:10.1016/j.lfs.2023.122352 38104863

[43] Asenjo-Bueno A, Alcalde-Estévez E, El Assar M, Olmos G, Plaza P, Sosa P, Martínez-Miguel P, Ruiz-Torres MP, López-Ongil S. 2021. Hyperphosphatemia-induced oxidant/antioxidant imbalance impairs vascular relaxation and induces inflammation and fibrosis in old mice. Antioxidants (Basel) 10:1308. doi:10.3390/antiox10081308 34439556 PMC8389342

[44] Hecker L, Logsdon NJ, Kurundkar D, Kurundkar A, Bernard K, Hock T, Meldrum E, Sanders YY, Thannickal VJ. 2014. Reversal of persistent fibrosis in aging by targeting Nox4-Nrf2 redox imbalance. Sci Transl Med 6:231ra47. doi:10.1126/scitranslmed.3008182 PMC4545252 24718857

[45] Abavisani M, Faraji S, Ebadpour N, Karav S, Sahebkar A. 2025. Beyond the Hayflick limit: how microbes influence cellular aging. Ageing Res Rev 104:102657. doi:10.1016/j.arr.2025.102657 39788433

[46] Shi J, Hao XY, Tong Y, Qian WB, Sun Y. 2024. SIRT6 alleviates senescence induced by Porphyromonas gingivalis in human gingival fibroblasts. Mol Biol Rep 51:976. doi:10.1007/s11033-024-09913-8 39259343

[47] Fu K, Cheung AHK, Wong CC, Liu W, Zhou Y, Wang F, Huang P, Yuan K, Coker OO, Pan Y, Chen D, Lam NM, Gao M, Zhang X, Huang H, To KF, Sung JJY, Yu J. 2024. Streptococcus anginosus promotes gastric inflammation, atrophy, and tumorigenesis in mice. Cell 187:882–896. doi:10.1016/j.cell.2024.01.004 38295787

