# Supplementary figures and images for: Fusobacterium nucleatum drives endothelial cell senescence by disrupting NOX4/NRF2 balance
Source: mBio. 2026 Jan 8;17(2):e03441-25. doi: 10.1128/mbio.03441-25 (PMC12892984; doi:10.1128/mbio.03441-25)

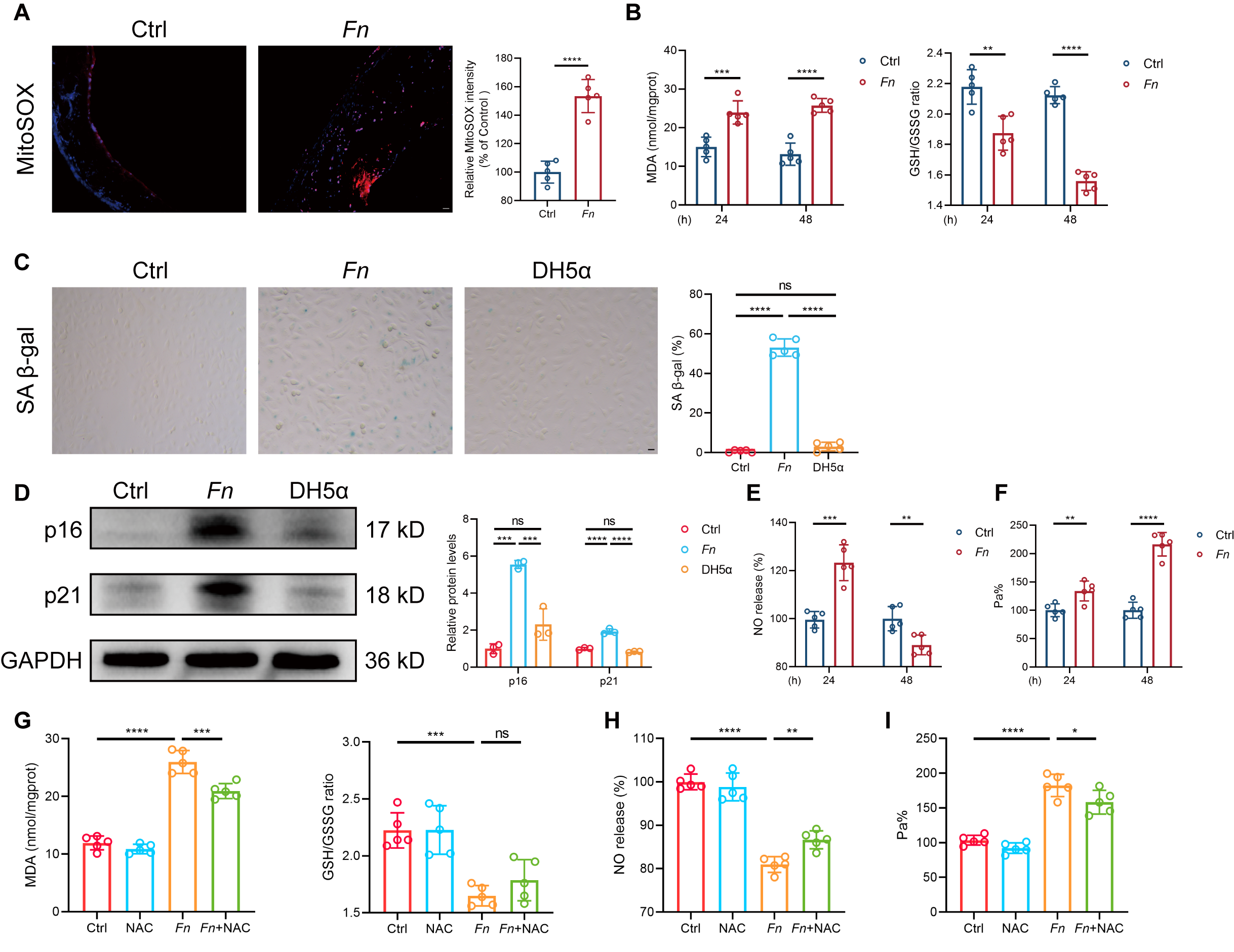

Supplement: Fig. S1 — Fn induced oxidative stress, senescence, and endothelial dysfunction in HUVECs. [file mbio.03441-25-s0001.tif]

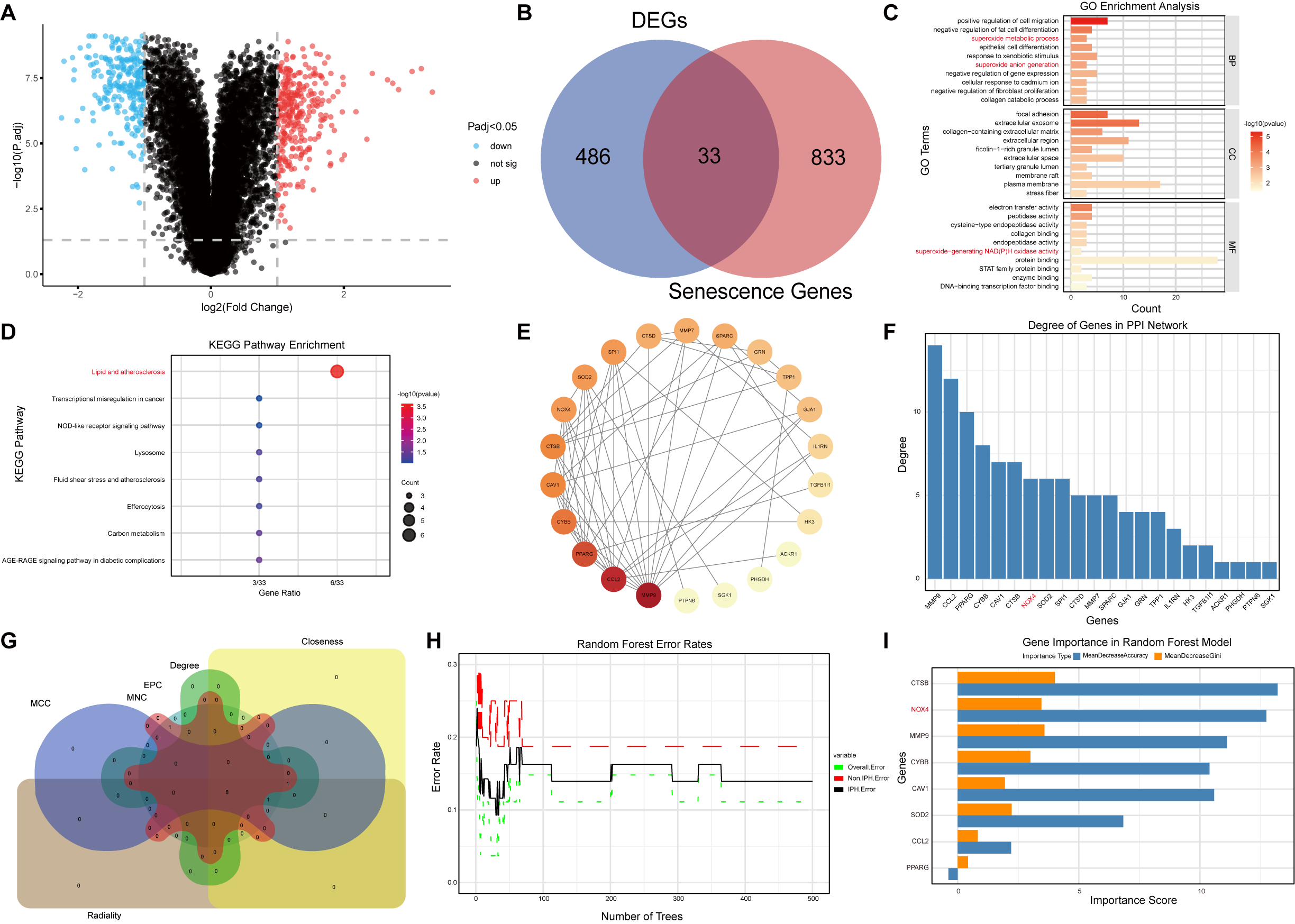

Supplement: Fig. S2 — Bioinformatics analysis identifies aging-related differential genes and key regulators in atherosclerosis. [file mbio.03441-25-s0002.tif]

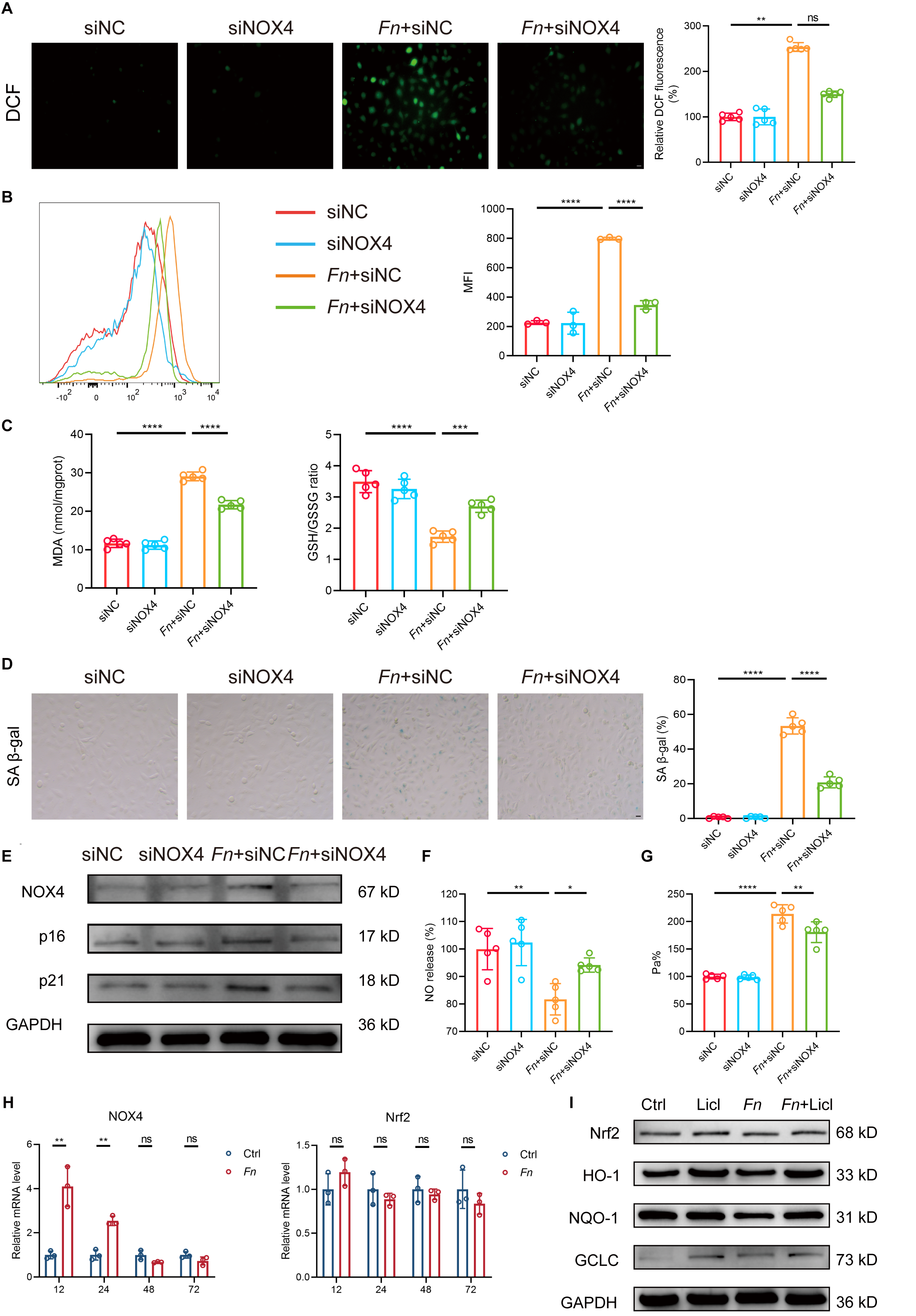

Supplement: Fig. S3 — NOX4 silencing and LiCl treatment modulate oxidative stress and endothelial function in Fn-infected HUVECs. [file mbio.03441-25-s0003.tif]

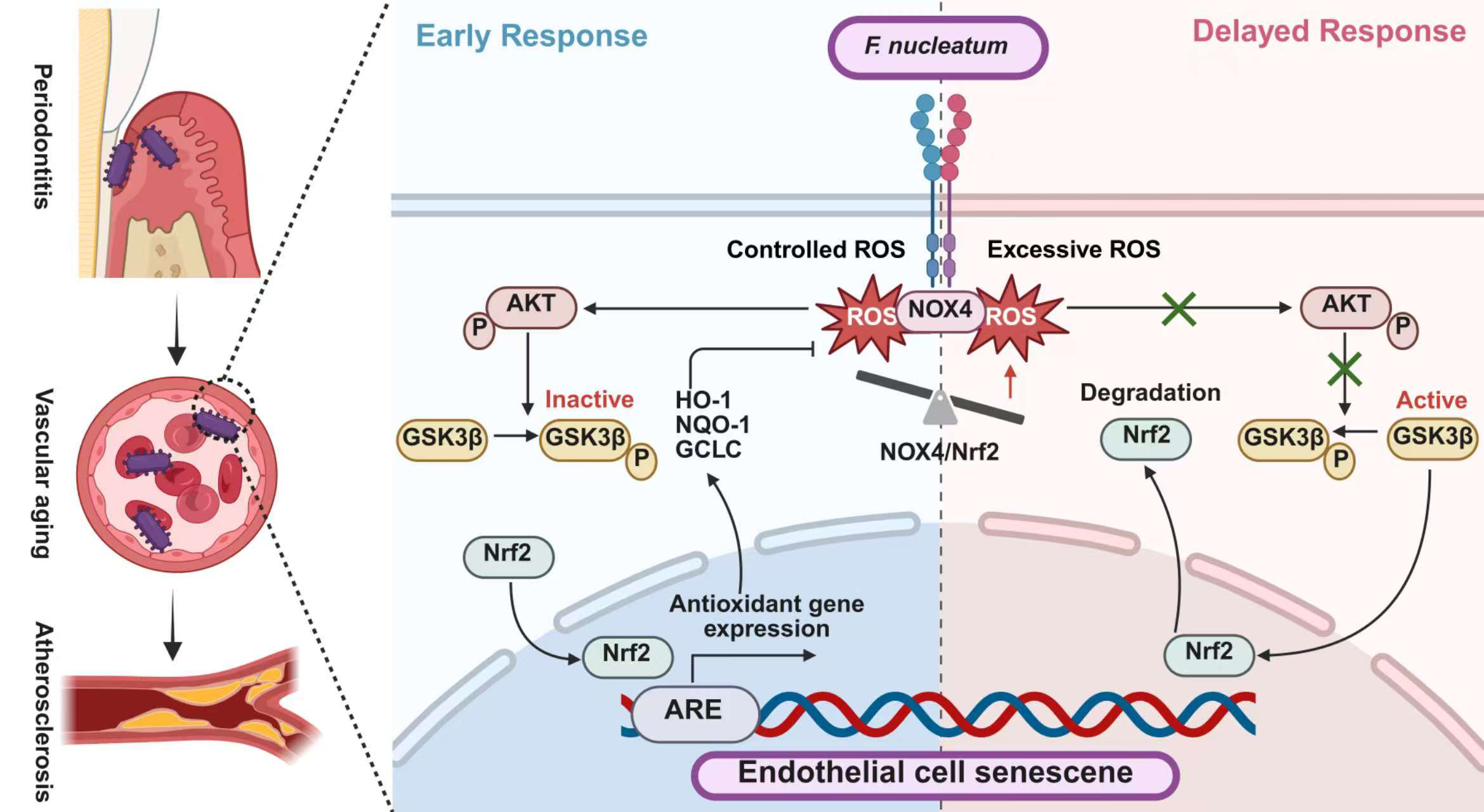

Supplement: Graphical Abstract — Summary. [file mbio.03441-25-s0004.tif]
