# Supplementary material for: Fusobacterium nucleatum drives endothelial cell senescence by disrupting NOX4/NRF2 balance
Source: mBio. 2026 Jan 8;17(2):e03441-25. doi: 10.1128/mbio.03441-25 (PMC12892984; doi:10.1128/mbio.03441-25)
Supplement: Legends — Supplemental figure legends. [file mbio.03441-25-s0005.docx]

**Appendix Materials**

***Fusobacterium nucleatum* drives endothelial cell senescence by disrupting NOX4/NRF2 balance**

Peiyao Wu, Jieyu Zhou, Jun Wang, Yafei Wu, Hui Wang*, Lei Zhao*

**Appendix Fig 1. *Fn* induced oxidative stress, senescence, and endothelial dysfunction in HUVECs**

(**A**) Representative fluorescence images of MitoSOX (red) and DAPI (blue) staining in aortic root sections from the Ctrl and *Fn* groups. Bar graph (right) shows the relative MitoSOX fluorescence intensity, normalized to the Ctrl group (n=5 samples/group). Scale bars = 20 µm. (**B**) Quantification of MDA levels (left) and the GSH/GSSG ratio (right) in HUVECs infected with *Fn* for 24 and 48 hours (n=5 samples/group). (**C**) Representative images (left) and quantification (right) of SA-β-gal staining in HUVECs (n=5 samples/group). Scale bar = 20 μm. (**D**) Representative western blot images (left) and quantification (right) of the protein expression levels of p16 and p21 in HUVECs (n=3 samples/group). GAPDH was used as a loading control. (**E**) Quantification of NO levels (n=5 samples/group) and (**F**) endothelial cell permeability (n=5 samples/group). (**G-I**) HUVECs were infected with *Fn* for 48 hours at MOI=100 with or without NAC. (**G**) Quantification of MDA levels (left) and the GSH/GSSG ratio (right) (n=5 samples/group). (**H**) Quantification of NO (n=5 samples/group) and (**I**) endothelial permeability (n=5 samples/group). Data are presented as mean ± SD. **p* < 0.05, ***p* < 0.01, ****p* < 0.001, *****p* < 0.0001. ns, not significant (Unpaired t-test for A-B, E-F, One-way ANOVA with Tukey’s multiple comparison test for C-D, G-I).

**Appendix Fig 2. Bioinformatics analysis identifies aging-related differential genes and key regulators in atherosclerosis.**

(**A**) Volcano plot showing DEGs in the GSE163154 dataset. (**B**) venn diagram of DEGs in GSE163154 and 866 aging-related genes from the Cell AGE database. (**C**) GO enrichment analysis of ARDEGs. (**D**) KEGG pathway analysis of ARDEGs. (**E**) PPI network of ARDEGs constructed using the STRING database. (**F**) Degree scores of the PPI network. (**G**) Venn diagram showing the intersection of core genes identified by six cytoHubba algorithms (MCC, MNC, Degree, Closeness, Radiality, and EPC) in Cytoscape. (**H**) Error rate curve of the random forest model. (**I**) Gene importance ranking in the random forest model based on both MDG and MDA metrics.

**Appendix Fig 3. NOX4 silencing and LiCl treatment modulate oxidative stress and endothelial function in *Fn*-infected HUVECs.**

HUVECs were infected with *Fn* for 48 hours at MOI=100 with or without siNOX4. (**A**) Representative fluorescence images (left) and quantification (right) of intracellular ROS levels (n=5 samples/group). Scale bar = 20 μm. (**B**) Representative histograms (left) and quantification (right) of ROS levels in HUVECs by FACS (n=3 samples/group). (**C**) Quantification of MDA levels (left) and the GSH/GSSG ratio (right) (n=5 samples/group). (**D**) Representative images (left) and quantification (right) of SA-β-gal staining in HUVECs (n=5 samples/group). Scale bar = 20 μm. (**E**) Western blot analysis of NOX4, p16 and p21 in HUVECs. GAPDH was used as a loading control. (**F**) Quantification of NO levels (n=5 samples/group) and (**G**) endothelial permeability in HUVECs (n=5 samples/group). (**H**) qRT-PCR analysis of NOX4 and NRF2 mRNA levels in HUVECs infected with *Fn* for 12, 24, 48, and 72 hours at an MOI of 100. (**I**) Western blot analysis of Nrf2, HO-1, NQO-1, and GCLC in HUVECs infected with *Fn* for 48 hours at MOI=100 with or without LiCl. GAPDH was used as a loading control. Data are presented as mean±SD. **p* < 0.05, ***p* < 0.01, ****p* < 0.001, *****p* < 0.0001. ns, not significant (Unpaired t-test for H, One-way ANOVA with Tukey’s multiple comparison test for A-G, I).
