# Supplementary material for: Fusobacterium nucleatum drives endothelial cell senescence by disrupting NOX4/NRF2 balance
Source: mBio. 2026 Jan 8;17(2):e03441-25. doi: 10.1128/mbio.03441-25 (PMC12892984; doi:10.1128/mbio.03441-25)

Western Blot - Uncropped Whole Membrane in Fig 1

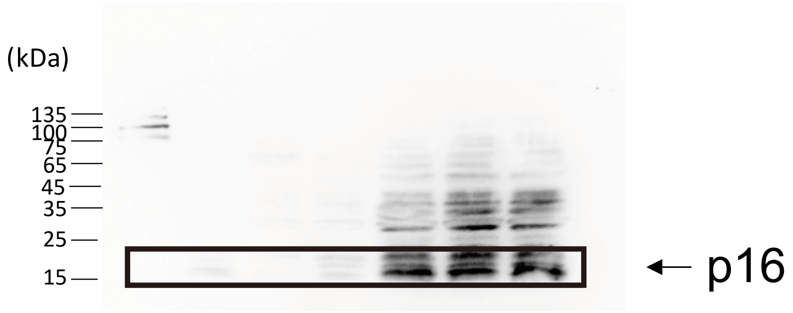

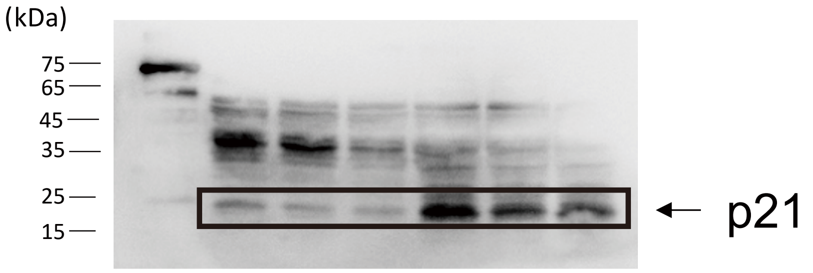

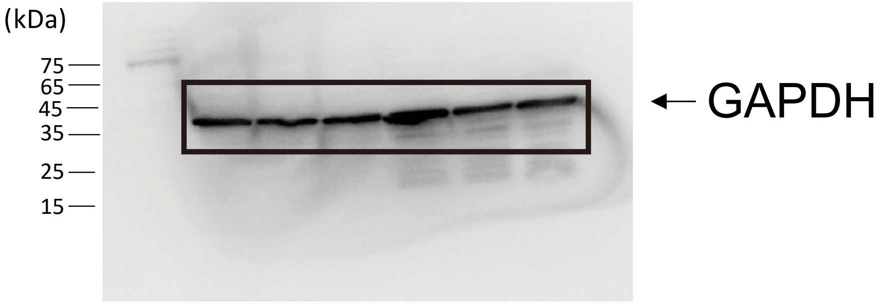

Western Blot - Uncropped Whole Membrane in Fig 2

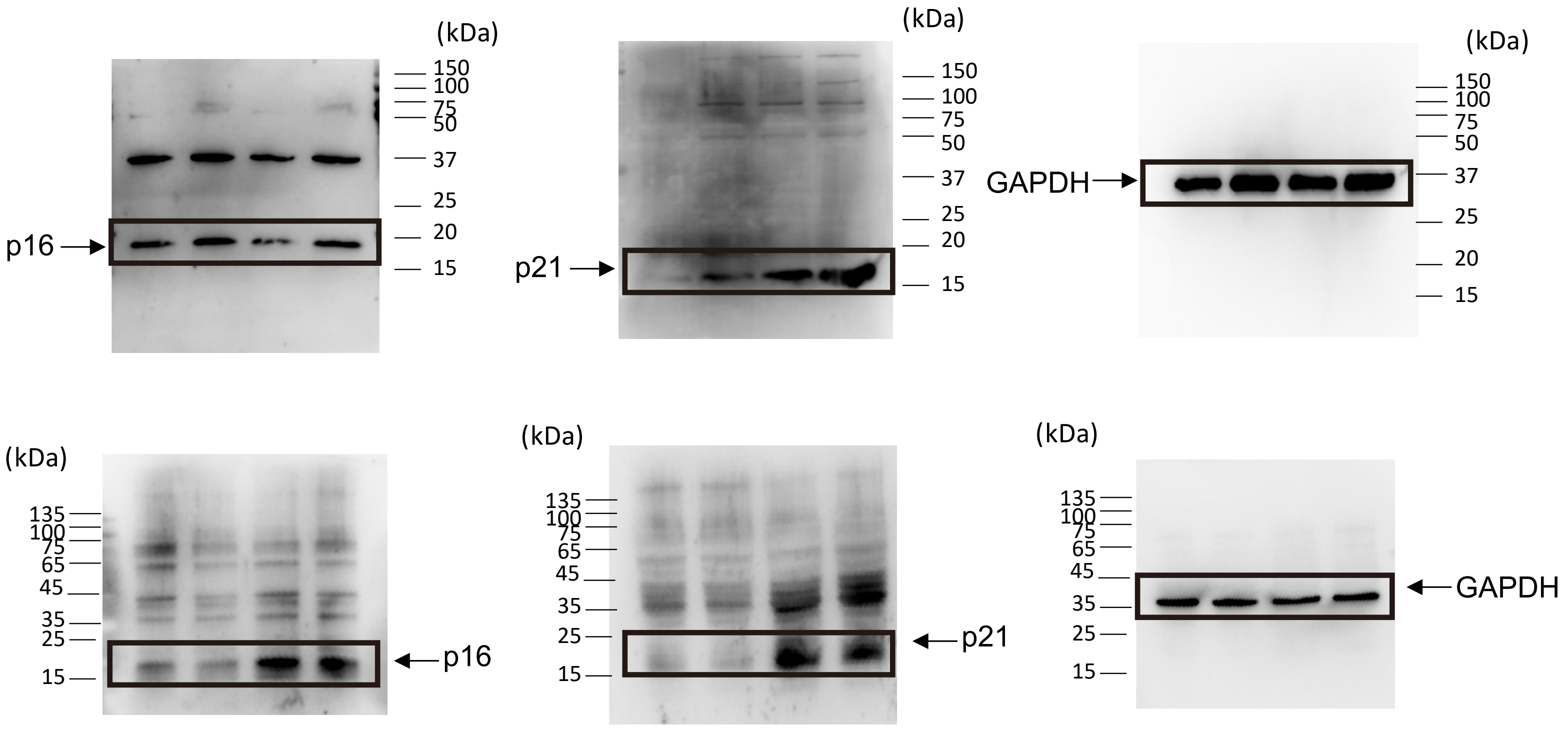

Western Blot - Uncropped Whole Membrane in Fig 3

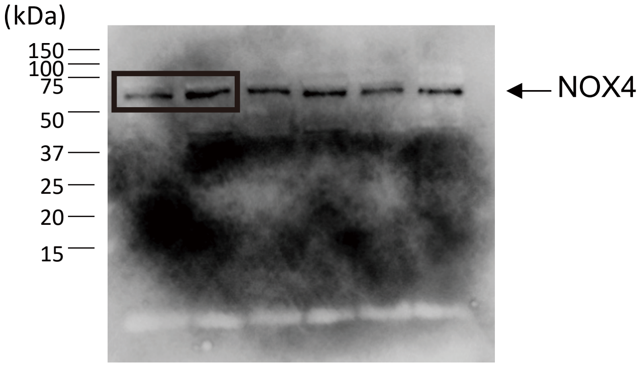

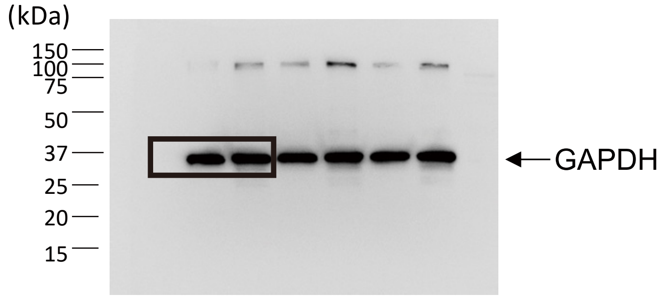

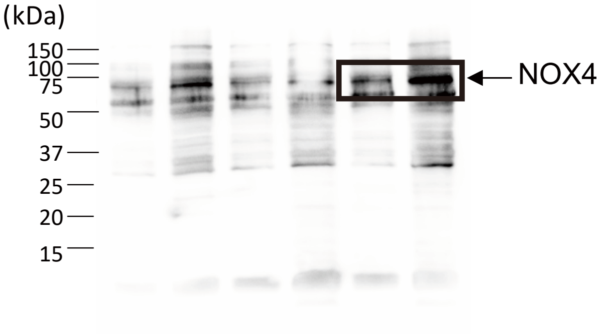

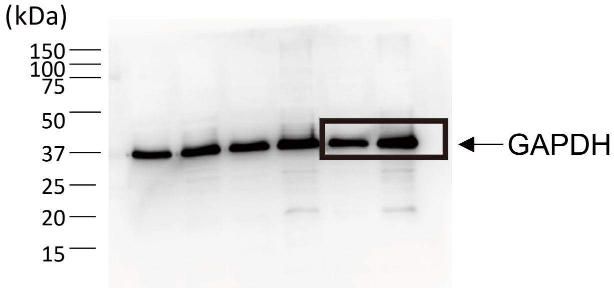

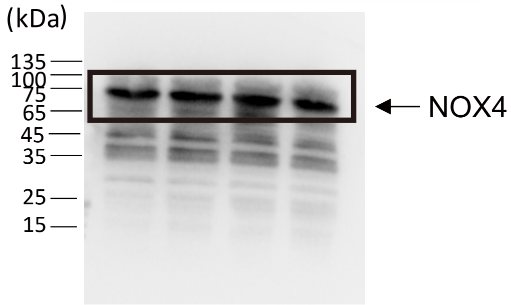

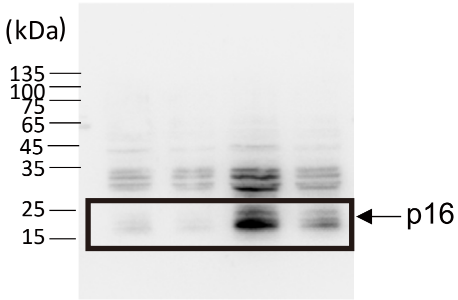

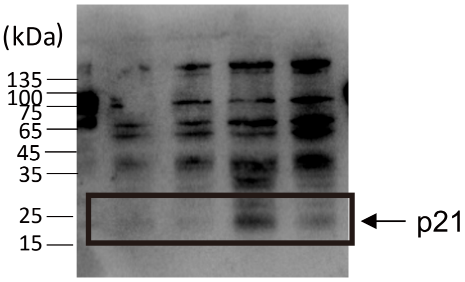

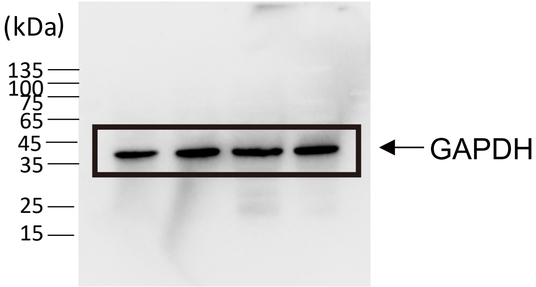

Western Blot - Uncropped Whole Membrane in Fig 4

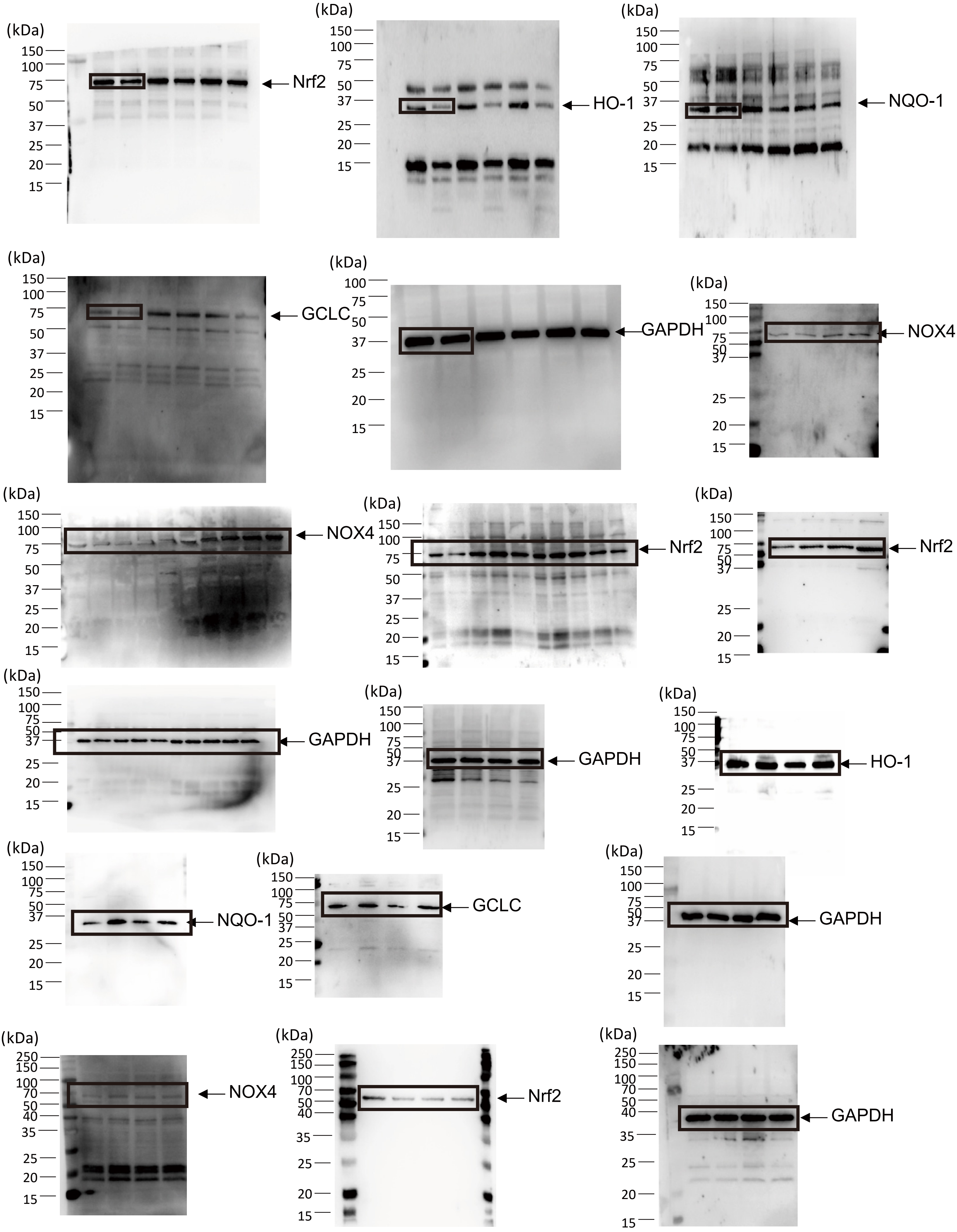

Western Blot - Uncropped Whole Membrane in Fig 4

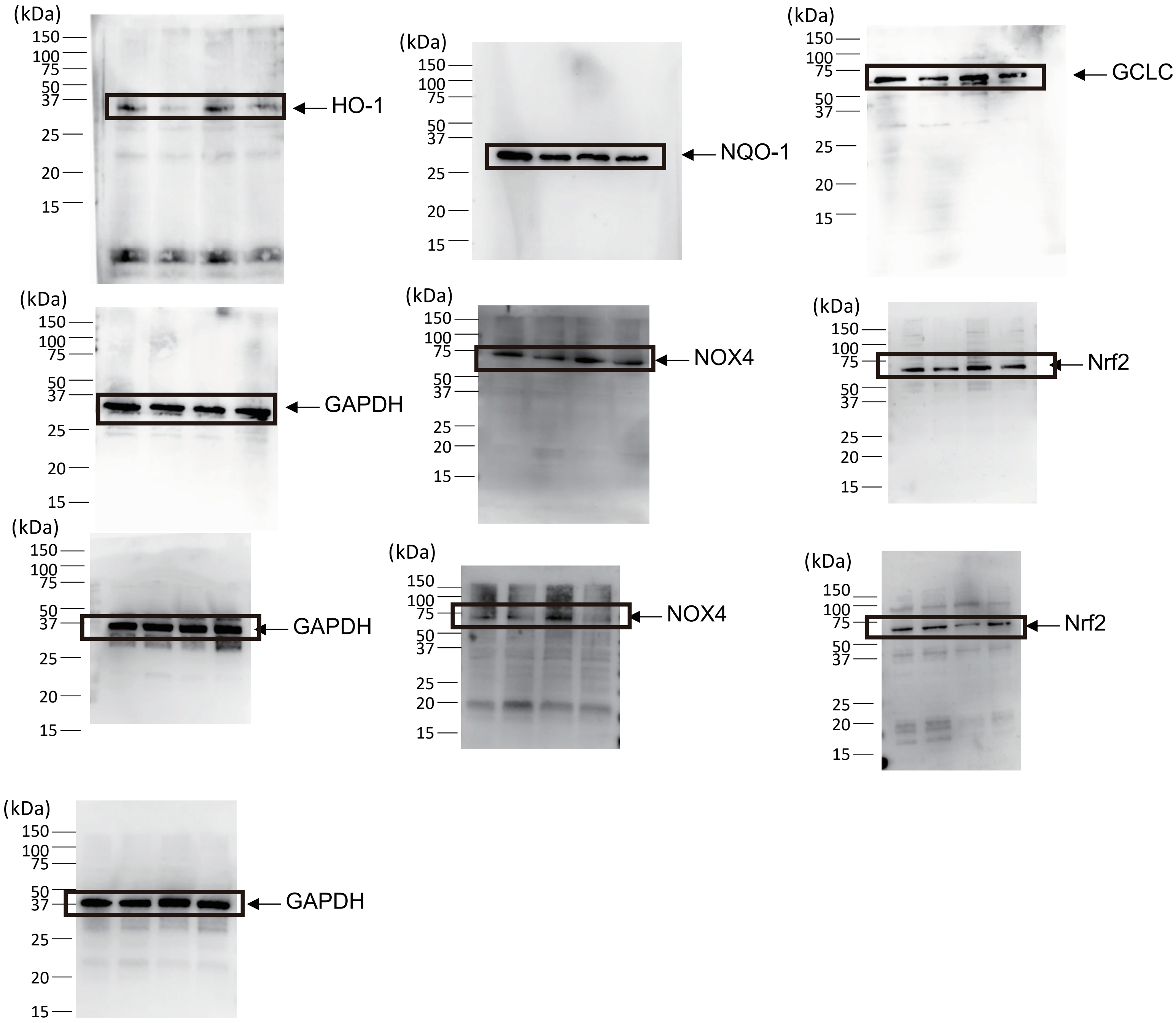

Western Blot - Uncropped Whole Membrane in Fig 5

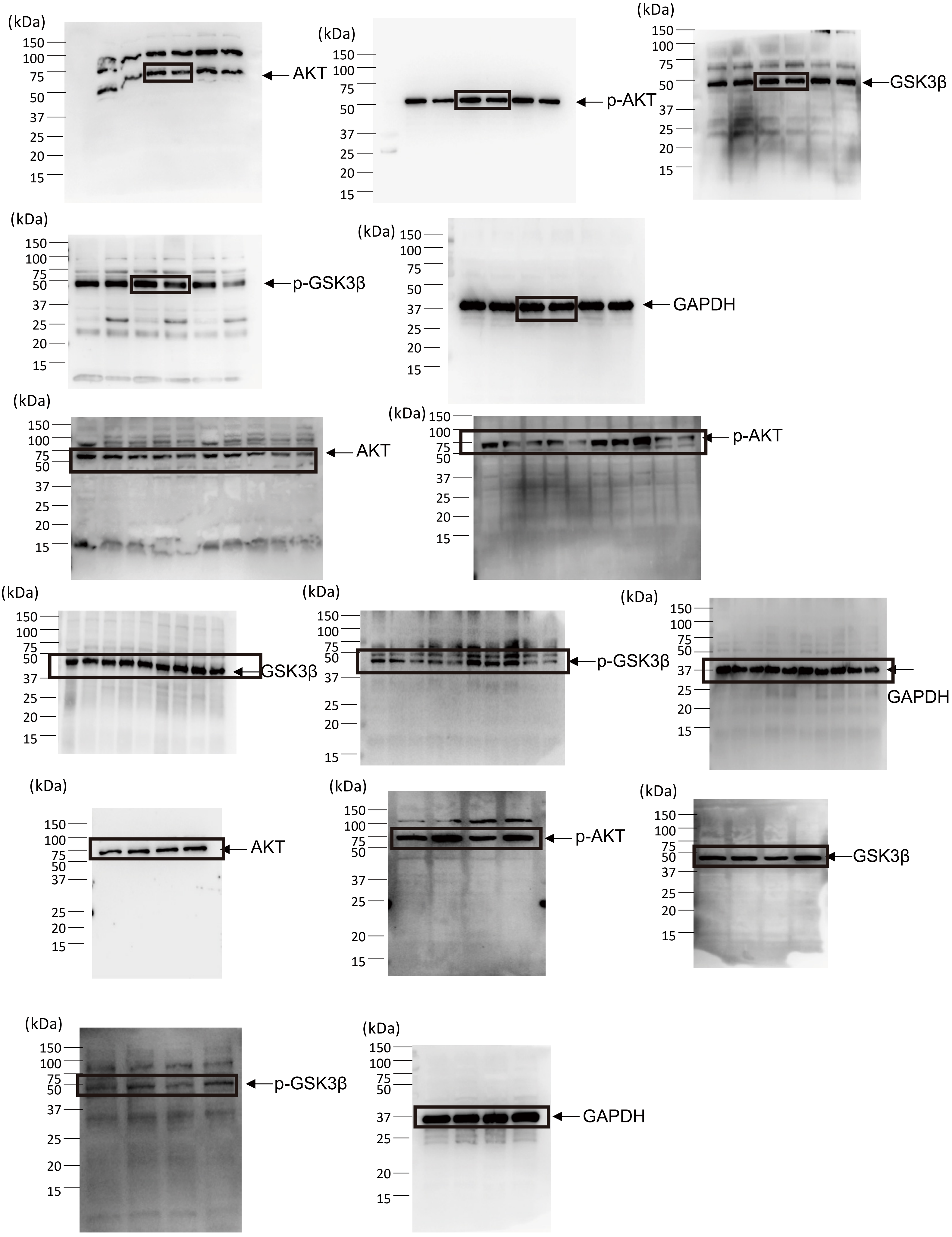

Western Blot - Uncropped Whole Membrane in Fig 5

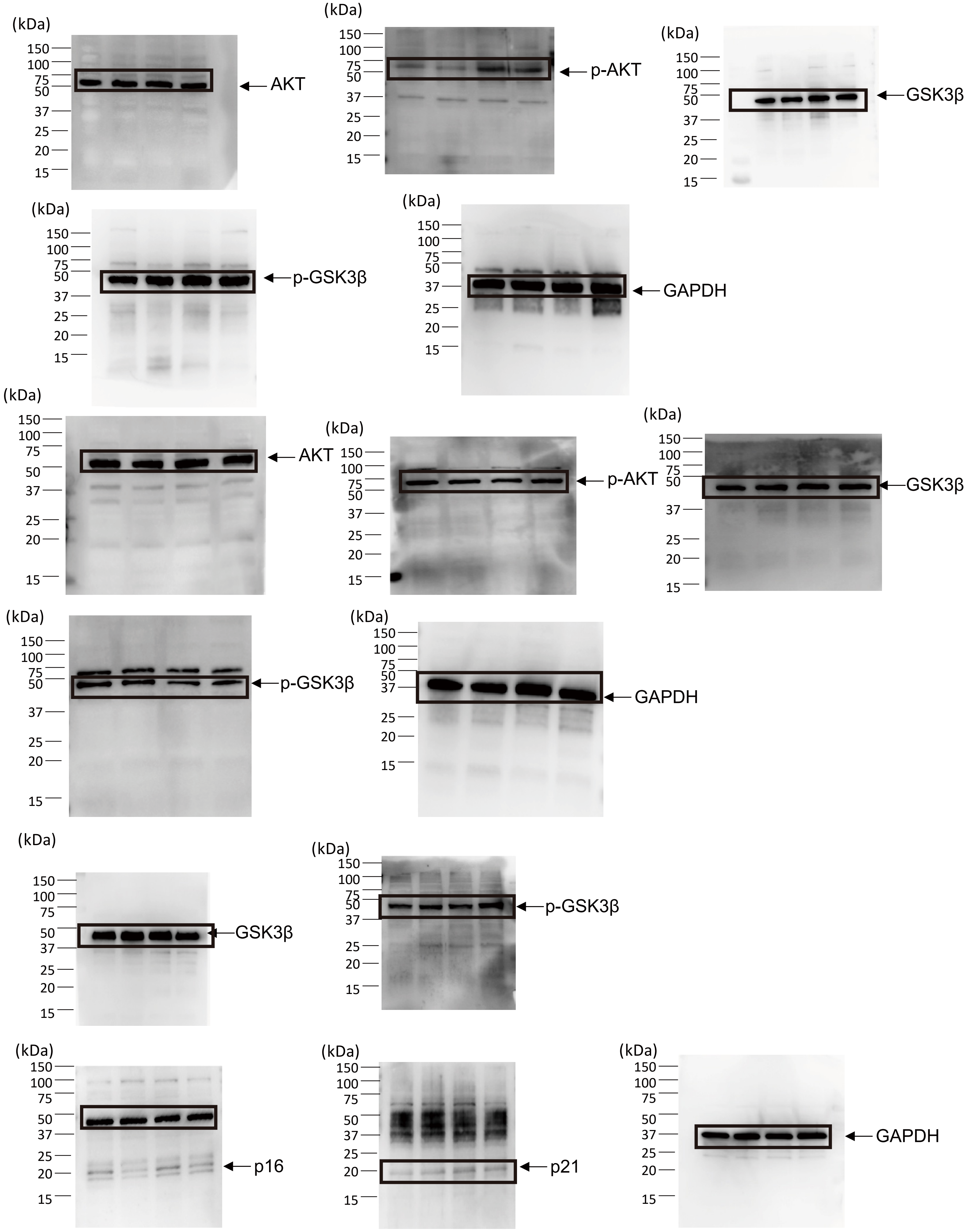

Western Blot - Uncropped Whole Membrane in Appendix Fig 1

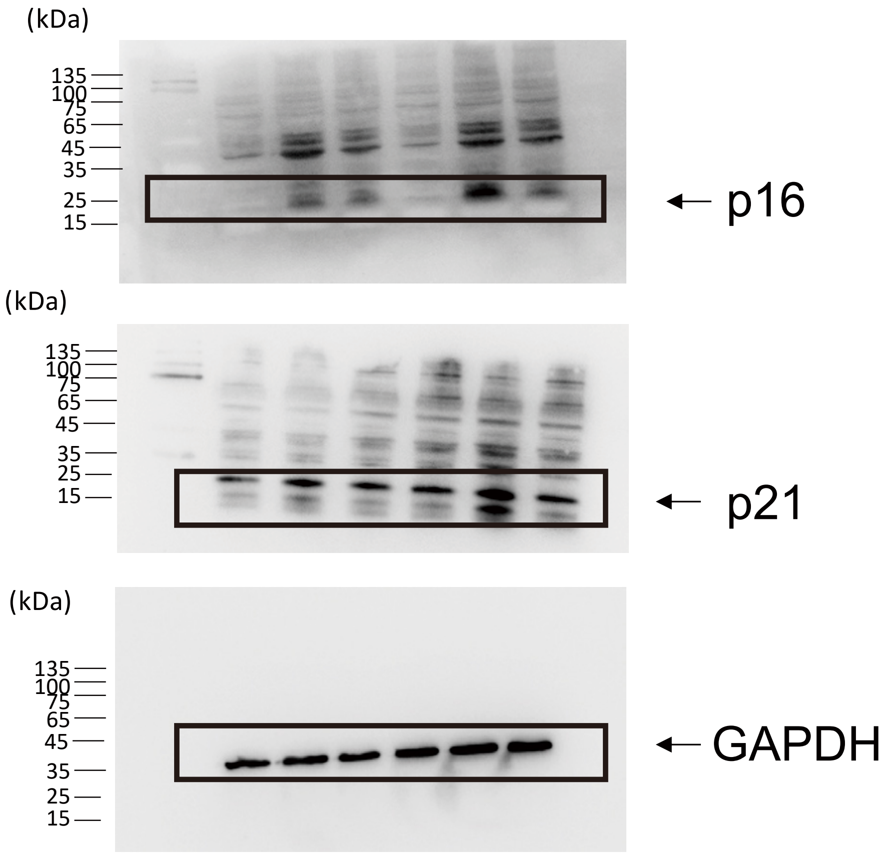

Western Blot - Uncropped Whole Membrane in Appendix Fig 3

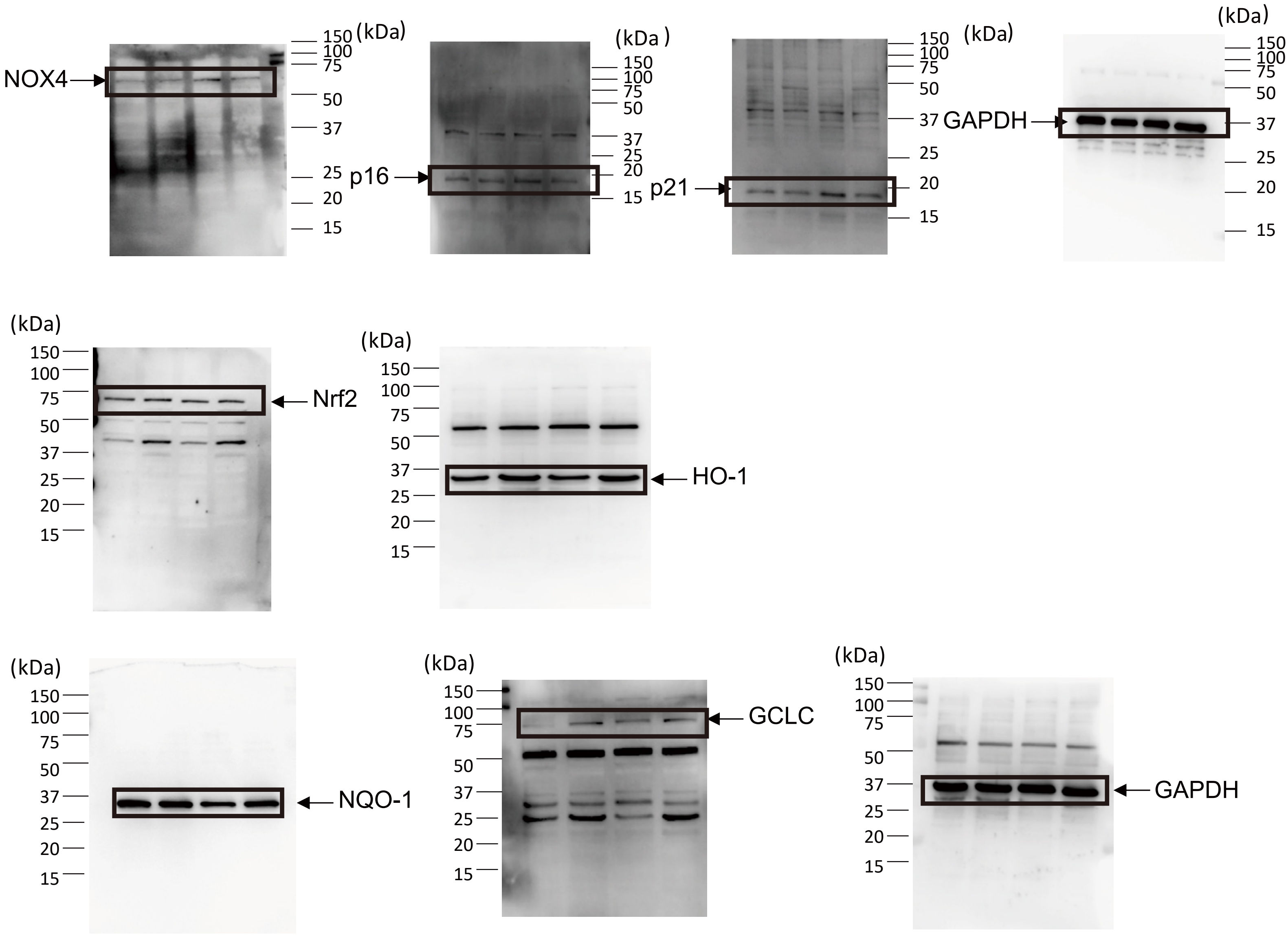

Supplement: Supplemental blots — Full uncropped WB membranes. [file mbio.03441-25-s0006.pdf]
