# Supplementary material for: Fusobacterium nucleatum drives endothelial cell senescence by disrupting NOX4/NRF2 balance
Source: mBio. 2026 Jan 8;17(2):e03441-25. doi: 10.1128/mbio.03441-25 (PMC12892984; doi:10.1128/mbio.03441-25)
Supplement: Table S1 — Primers. [file mbio.03441-25-s0007.docx]

**Appendix Table 1 The primers used in this study.**

| Name | Sense primer (5′→3′) | Antisense primer (5′→3′) |
| --- | --- | --- |
| siRNA |  |  |
| siNOX4 | CCGUUGGUUUGCAGAUUUATT | UAAAUCUGCAAACCAACGGTT |
| siNC | UUCUCCGAACGUGUCACGUTT | ACGUGACACGUUCGGAGAATT |
| qRT-PCR |  |  |
| NOX4 | CAGAAGGTTCCAAGCAGGAG | GTTGAGGGCATTCACCAGAT |
| Nrf2 | ATATTCCCGGTCACATCGAGA | ATGTCCTGTTGCATACCGTCT |
| GAPDH | ACCCAGAAGACTGTGGATGG | AGGCCATGCCAGTGAGCTT |
